# Marine vs. freshwater cyanobacteria as biostimulants for wheat under salt stress: morphophysiological, anatomical and phytochemical perspectives

**DOI:** 10.1186/s12870-025-07353-5

**Published:** 2025-10-02

**Authors:** Rania M. Mahmoud, Mostafa M. El-Sheekh, Amr ElKelish, Asmaa A. Adawy, Ahmed A.M. Yassein, Ibrahim A.A. Mohamed, Ayaat A. Teleb

**Affiliations:** 1 Botany Department, Faculty of Science, Fayoum University, Fayoum, 63514 Egypt; 2 Botany Department, Faculty of Science, Tanta University, Tanta, 31527 Egypt; 3 Department of Biology, College of Science, Imam Mohammad Ibn Saud Islamic University (IMSIU), Riyadh, 11623 Saudi Arabia; 4 Genetic Department, Faculty of Agriculture, Fayoum University, Fayoum, 63514 Egypt; 5 Botany Department, Faculty of Agriculture, Fayoum University, Fayoum, 63514 Egypt

**Keywords:** Cyanobacterial biostimulants, Salt stress tolerance, Wheat genotypes, Root anatomy, Phytohormones and bioactives

## Abstract

Salinity is a major abiotic stress that restricts global crop productivity, particularly in staple cereals such as wheat (*Triticum aestivum*). This study investigates the biostimulant potential of two cyanobacterial strains, *Alkalinema pantanalense* (freshwater origin) and *Geminocystis* sp. (marine origin), on the early growth of three wheat genotypes under varying salt stress conditions (0, 50, 100, and 200 mM NaCl). Algal extracts were applied to seeds and seedlings, and physiological, anatomical, and biochemical responses were assessed. Results revealed that *A. pantanalense* significantly improved seed germination and chlorophyll content under moderate salinity (50–100 mM), especially in genotype G2, with shoot length reaching up to 3.5 cm and chlorophyll levels maintained near control values. In contrast, *Geminocystis* sp. enhanced root dry weight even under high salinity (200 mM), suggesting improved osmotic adjustment, though it reduced root length and meristem size in some genotypes. Anatomical analysis revealed that *A. pantanalense* increased the number of root meristem cells and promoted vascular differentiation, thereby supporting structural resilience. Phytochemical profiling revealed that *A. pantanalense* was richer in flavonoids (130.7 µg/g naringin) and phenolic acids (ferulic and caffeic), while *Geminocystis* sp. had higher phytohormone levels, notably IAA (20.95 µg/g ) and BA. GC-MS analysis identified bioactive compounds such as lupeol and oleic acid in *A. pantanalense*, and phytol and methyl esters in *Geminocystis* sp. These findings demonstrate distinct yet complementary biostimulant profiles between the two cyanobacteria, underscoring their potential for sustainable wheat cultivation in saline soils.

## Introduction

Soil salinity is a widespread and escalating threat to global agricultural productivity, affecting approximately 20% of cultivated land and expected to expand further due to climate change, unsustainable irrigation practices, and the use of saline water sources [1–3]. Salinity impairs plant growth through osmotic stress, ion toxicity, and oxidative damage, ultimately reducing yields and threatening food security [4, 5]. Traditional mitigation approaches such as breeding salt-tolerant cultivars and optimizing irrigation are often time-consuming, costly, and limited in scalability [6, 7] Consequently, there is growing interest in biological interventions such as microbial biostimulants, particularly cyanobacteria, which offer sustainable and multifaceted mechanisms for enhancing plant tolerance to salinity stress [5, 8–10].

Cyanobacteria, often referred to as blue-green algae, are a phylogenetically diverse group of photosynthetic prokaryotes that play a critical role in global ecosystems. Their ability to perform oxygenic photosynthesis and fix atmospheric nitrogen has made them key contributors to the carbon and nitrogen cycles [11]. Found in a variety of habitats from freshwater lakes to marine waters and extreme environments like deserts and saline soils, cyanobacteria exhibit remarkable adaptability, which is attributed to their production of bioactive compounds [12, 13]. Historically, cyanobacteria have been utilized in agriculture as biofertilizers, particularly in rice paddies, where nitrogen-fixing species like Anabaena and Nostoc enhance soil fertility [14, 15]. However, their role has expanded beyond nitrogen fixation in recent years, with growing evidence of their potential as biostimulants [16, 17]. Biostimulants are defined as substances or microorganisms that improve plant growth, nutrient efficiency, and stress tolerance without directly supplying nutrients or controlling pests [18, 19]. Cyanobacteria fit this definition by producing a suite of bioactive molecules, including phytohormones, exopolysaccharides (EPS), amino acids, peptides, and antioxidants, which collectively enhance plant performance under adverse conditions [15, 20].

The biostimulant effects of cyanobacteria are mediated through various mechanisms, each associated with specific bioactive compounds. Phytohormones, including auxins, cytokinins, and gibberellins, are among the most researched [21]. Auxins promote root development and branching; cytokinins stimulate cell division and delay senescence; and gibberellins enhance stem elongation and seed germination. Studies have shown that cyanobacteria like Spirulina and Anabaena produce these hormones, resulting in improved plant growth when used as Biostimulants [23]. Exopolysaccharides (EPS) are high-molecular-weight polysaccharides secreted by cyanobacteria that serve a dual purpose in soil and plant health. In the soil, EPS improves structure by promoting aggregation, which enhances water retention and aeration. For plants, EPS can mitigate salinity stress by binding Na⁺ ions in the rhizosphere, thereby reducing their uptake and toxicity [15, 25]. Recent research has highlighted the EPS-producing abilities of cyanobacteria, such as Nostoc and Phormidium, emphasizing their potential in saline agriculture. Amino acids and peptides, another category of cyanobacterial metabolites, act as nitrogen sources and osmoprotectants [28]. Compounds like proline and glycine betaine accumulate in cyanobacteria under stress and, when applied to plants, help maintain cellular osmotic balance and protect against dehydration [15]. Additionally, these molecules can serve as signaling agents, triggering defense responses that enhance stress tolerance. Antioxidants, including carotenoids, tocopherols, and phenolic compounds, are produced by cyanobacteria to combat oxidative stress in their cells. When applied to plants, these antioxidants scavenge ROS generated under salinity stress, protecting cellular integrity and sustaining metabolic functions. Recent studies have identified high antioxidant activity in cyanobacterial extracts from *Synechococcus* and *Microcystis*, indicating their potential for stress mitigation [27, 33].

This research investigates two cyanobacteria isolates: *Geminocystis* sp., isolated from a marine habitat, and *Alkalinema pantanalense*, sourced from a freshwater environment. These isolates were selected for their resilience in harsh conditions, suggesting they may harbor unique biostimulant properties suited to salinity stress mitigation. *Geminocystis* sp. belongs to the Geminocystaceae family, characterized by unicellular or colonial forms [34]. Its marine origin provides a baseline for comparing biostimulant effects with freshwater isolates. *Alkalinema pantanalense*, was first isolated from Pantanal wetlands in Brazil [35]. Freshwater cyanobacteria like *Geminocystis *sp. often exhibit enhanced production of osmoprotectants and antioxidants due to their natural habitat, making them promising candidates for salt-stress alleviation [36, 37]. The ecological contrast between these isolates enables a robust comparison of freshwater versus marine cyanobacteria in agricultural applications.

Wheat (*Triticum aestivum *L.) is a cornerstone of global food security, contributing approximately 20% of the calories consumed by humans [38]. However, its moderate sensitivity to salinity yields declines significantly above 6 dS/m, posing a major challenge in salt-affected regions [39]. Salinity impacts wheat at all growth stages, reducing germination rates, root development, and grain yield [40]. Given its economic and nutritional importance, improving wheat’s salt tolerance is a priority for sustainable agriculture. In this study, three wheat genotypes were selected to assess genotypic variation in response to cyanobacterial biostimulants. This approach accounts for genetic diversity, which can influence the efficacy of biostimulant treatments [41, 42].

Given the increasing need for effective and sustainable biostimulants, cyanobacteria have gained attention due to their ability to produce diverse bioactive compounds such as phytohormones, exopolysaccharides, flavonoids, and phenolic acids that contribute to plant growth and stress resilience. However, the performance of cyanobacterial biostimulants under salinity stress may vary depending on their ecological origin and metabolite profiles. In this study, we selected two taxonomically and ecologically distinct cyanobacterial strains: the freshwater-derived *Alkalinema pantanalense,* and the marine water *Geminocystis* sp., which offers a comparative baseline for evaluating salt tolerance enhancement.

The present work aims to: (1) characterize the bioactive phytochemical composition of these cyanobacterial extracts, including phytohormones, phenolics, and flavonoids; (2) evaluate their effects on morphophysiological and anatomical traits in three wheat (*Triticum aestivum* L.) genotypes under graded salt stress; and (3) determine whether differences in ecological origin correlate with biostimulant efficacy. Through this approach, we seek to advance the application of cyanobacterial bioproducts in saline agriculture and identify strain-specific mechanisms that promote salt stress tolerance.

## Materials and methods

### Algal isolation and molecular identification

The algal strains were obtained from several water habitats in Egypt’s Fayoum Governorate. *A. pantanalense* and *Geminocystis* sp. were isolated from freshwater Bahr Yousef (29.2756798 N, 30.8226603 E) and brackish water Lake Qarun, near the mouth outlet of Elbatts drain (30.8435, 29.5039), respectively. Both strains were cultured under laboratory-controlled conditions. Specifically, the cultures were maintained for 21 days in Zarrouk medium for *Geminocystis* (pH 9) and MBL medium for *Alkalinema* (pH 7.5), under a temperature of 25 ± 1 °C, light intensity of 40 µmol photons m⁻² s⁻¹, and a photoperiod of 12:12 h light: dark, no CO₂ supplementation or aeration was applied for either strain during cultivation. Primers WAW8F and WAW23SR were used to amplify the 16 S rRNA gene in order to perform molecular identification [43] (Table 1). Sequencing of the amplified products was done for verification.

**Table 1:** Primers used in this study

Name	Sequence
WAW8F	AGAGTTTGATCCTGGCTCAG
WAW23SR	TTTGCGGCCGCTCTGTGTGCCTAGGTATCC

To do phylogenetic analysis on the amplified sequences, MEGA version 11’s Maximum Likelihood (ML) technique was selected. In order to evaluate the robustness of the inferred phylogenetic linkages, 1,000 bootstrap replications were performed. The Kimura 2-parameter model was shown to be the best-fit model of nucleotide substitution [44, 45]. The 16 S rRNA gene sequences obtained during this study were included in the NCBI GenBank database under accession numbers OR610313 for strain *Geminocystis* sp. and OR469049 for strain RMFY20 of *Alkalinema pantanalense*.

### Algal extract Preparation

With fresh algal culture, a water-based extract was made in order to evaluate the impact of algal extract on wheat (*Triticum aestivum* L.) under salinity stress. During the exponential growth phase, the culture was harvested at the end of the log phase with cell numbers (5428 ± 115) × 10^4^/ml for *Alkalinema* and 6062± 29 × 10^4^/ml for *Geminocystis* sp. by centrifugation at 5000 rpm for 10 min. Any remaining medium components were then removed by washing with distilled water. After the biomass was suspended in distilled water at a 1:10 (w/v) ratio, it was ultrasonically homogenized for 15–20 min using a probe-type ultrasonic processor (20 kHz, 400 W). This procedure facilitated the bursting of algal cells and the release of intracellular bioactive substances. The homogenate was used as the crude algal extract (1gram dry weight). The extract was applied either alone or in conjunction with varying salt concentrations to irrigate the wheat genotypes.

### Analysis of algal extract

The following analysis procedures were applied to ethanolic algal extracts in order to determine each extract’s chemical components.

#### HPLC analysis

##### Plant hormones

The HPLC Isocratic separation was achieved using a C18 reversed-phase column (150 × 4.6 mm i.d.; 5 μm) and the mobile phase Acetonitrile and acidic water (0.01% H_3_PO_4_) in the ratio of 60:40. Separations were carried out at room temperature with a flow rate of 1 mL/min and an injection loop of 20 µL. analysis was performed at 206 nm. All values represent the quantitation in 1-gram dry weight of each algal isolate.

##### Phenolic and flavonoids

Thermo Fisher Scientific’s VanquishTM Core HPLC apparatus, which includes an auto-sampling injector, solvent degasser, two LC-pumps (series 1100), ChromeleonTM Chromatography Data System (CDS), and UV/Vis. detector (set at 250 nm for phenolic acids and 360 nm for flavonoids), was used to analyze phenolic and flavonoid compounds. The C18 column (125 mm × 4.60 mm, 5 μm particle size) was used for the analysis. A gradient mobile phase consisting of two solvents—Solvent A (methanol) and Solvent B (acetic acid in water (1:25), was utilised to separate phenolic acids. For the first three minutes of the gradient procedure, the concentration was maintained at 100% B. For the next five minutes, 50% eluent A was used. Then, for the next two minutes, the concentration of A was raised to 80%, and for the next five minutes, it was lowered to 50% once more at a detection wavelength of 250 nm. Using an isocratic elution (70:30) procedure, flavonoids were separated using a mobile phase consisting of two solvents: acetonitrile (A) and 0.2% (v/v) aqueous formic acid (B). The separation was carried out at 25 °C with a solvent flow rate of 1 ml/min. 25 µL was the injection volume. All values represent the quantitation in 1-gram dry weight of each algal isolate.

#### Gas chromatography–mass spectrometry (GC-MS) analysis

Using a direct capillary column TG–5MS (30 m x 0.25 mm x 0.25 μm film thickness) and a GC-TSQ mass spectrometer (Thermo Scientific, Austin, TX, USA), the chemical composition of the samples was determined. The temperature of the column oven was first maintained at 60 °C, then raised by 5 °C per minute to 250 °C with a 2-minute hold, and finally raised to 300 °C with 30 °C per minute. At 270 °C, the injector temperature was maintained. At a steady flow rate of 1 millilitre per minute, helium was employed as a carrier gas. Diluted samples of 1 µl were automatically injected using the Autosampler AS3000 paired with GC in the split mode after a 4-minute solvent delay. In full scan mode, EI mass spectra were obtained at 70 eV ionization voltages across the m/z 50–650 range. The temperature of the transfer line and ion source was set at 280 °C and 200 °C, respectively. By comparing the components’ mass spectra to those of the NIST14 and WILEY 09 mass spectral databases, the components were identified.

### Plant materials

To evaluate the effectiveness of algal treatments in improving wheat (*Triticum aestivum L*.) seed tolerance to salt stress, a seedling experiment was carried out at the Faculty of Agriculture, Fayoum University (Fayoum, Egypt). Three wheat genotype seeds, Misr3 (G1), Sids14 (G2), and Misr1 (G3), were generously provided by the Fayoum University Faculty of Agriculture’s Department of Agronomy.

Sterile Petri dishes containing ten seeds each were used for the experiment, with each treatment having three duplicates. Under carefully monitored laboratory conditions, the seeds were nurtured for seven days. Aqueous extracts of two algae species, *Geminocystis* sp. and *Alkalinema pantanalense*, produced as previously described, as well as four different doses of sodium chloride (NaCl) (0, 50, 100, and 200 mM), were used as treatments. Throughout the experiment, seeds received irrigation with the corresponding salt concentration as necessary to maintain the proper moisture levels.

To assess the combined effects of salinity and algal application on early seedling growth, the germination percentage, shoot and root lengths, and dry biomass were measured for each treatment after the incubation period.

### Experimental design

To assess the combined impacts of salt concentration, algal extract treatment on wheat genotypes performance, a factorial experiment was carried out. A completely randomized design (CRD) was used in the trial, and each treatment had three replications. Based on their known variations in salt tolerance, three wheat genotypes (G1, G2, and G3) were chosen. In addition to a control group that received no extract, two cyanobacterial extracts, *Alkalinema pantanalense* and *Geminocystis* sp. were employed as biostimulant treatments. Four concentrations of salt stress were applied: 0, 50, 100, and 200 mM NaCl.

The entire factorial arrangement of the factors genotype (3 levels), algal extract (3 levels: control, *Alkalinema pantanalense*, *Geminocystis* sp.), and salt concentration (4 levels) led to the experiment’s 36 treatment combinations. Three separate replicates of each treatment were used. At the initiation of the experiment, each Petri dish received a total of 10 mL of treatment solution, consisting of 5 mL of the respective NaCl concentration and 5 mL of the assigned algal extract.To maintain treatment consistency, the same volumes were reapplied after three days.

As needed to meet the goals of the study, information was gathered on the percentage of germination, root and shoot lengths, root and shoot dry weight, chlorophyll content and root tip anatomy. Three -way ANOVA was conducted to evaluate the interaction between algae treatments and salt concentration inside each genotype, followed by post-hoc Tukey HSD test to assess pairwise differences.

### Chlorophyll content and fluorescence measurements

A SPAD-502 chlorophyll meter (Minolta, Japan) was used to measure the relative chlorophyll content of juvenile leaves. A Handy PEA portable fluorometer (Hansatech Instruments Ltd., UK) was used to measure the fluorescence of chlorophyll in accordance with the approach outlined by Rady et al. (2021) [46, 47].

### Anatomical study

For observation of root tip anatomy, samples were taken two days after sowing (2 mm of root tips) fixed in FAA solution (containing 50 ml of 95% (v/v) ethanol + 10 ml of formaldehyde + 5 ml of glacial acetic acid + 35 ml of distilled water) for 12 h [48]. Thereafter, the samples were washed in 50% ethanol, dehydrated, and cleared in tertiary butanol series, and embedded in paraffin wax. Cross sections, 10 μm thick, were cut by a rotary microtome (Zhejiang Jinhua Kedi Instrument Equipment Co., Ltd., China), adhered with a Haupt’s adhesive, stained with a crystal violet erythrosin combination [49, 50], cleared in carbol xylene, and mounted in Canada balsam. The sections were observed using an upright light microscope (AxioPlan, Zeiss, Jena, Germany). CaseViewer 2.3 software program (3DHISTECH Ltd.) was used for image analysis and to perform measurements [51]. Root meristem size was expressed (According to [52]) as the distance between the quiescent center (QC, black arrows) and the transition zone (TZ; indicating the position of the first elongating cortical cell (blue arrows)) The root meristem number was represented by the number of meristematic cortex cells from the quiescent center (QC, black arrows) and the transition zone (TZ; blue arrows), which was counted as described by [53]. The average of root cap area was expressed as the area of the root cap (RC). Root tip diameter was measured at the transition zone (TZ) according to [54].

### Statistical analysis

Statistical analyses were performed using R programming (Version 4.3.3). Three-way ANOVA was used to assess treatment effects, followed by Tukey HSD multiple range test to evaluate mean differences at the 5% significance level (*P* < 0.05) for growth parameters and Duncan for anatomical studies. Phylogenetic analysis was conducted using MEGA version 11. Principal component analysis (PCA), heatmap correlations, and boxplots were generated in R (version 4.3.3) using the ggstatsplot and other relevant packages [55].

## Results

### Identification of algal strains

The identification of the two algal isolates was performed using molecular techniques targeting the 16 S region. The isolates exhibited a resemblance of 99–100% with their closely related species.

**Fig. 1:**
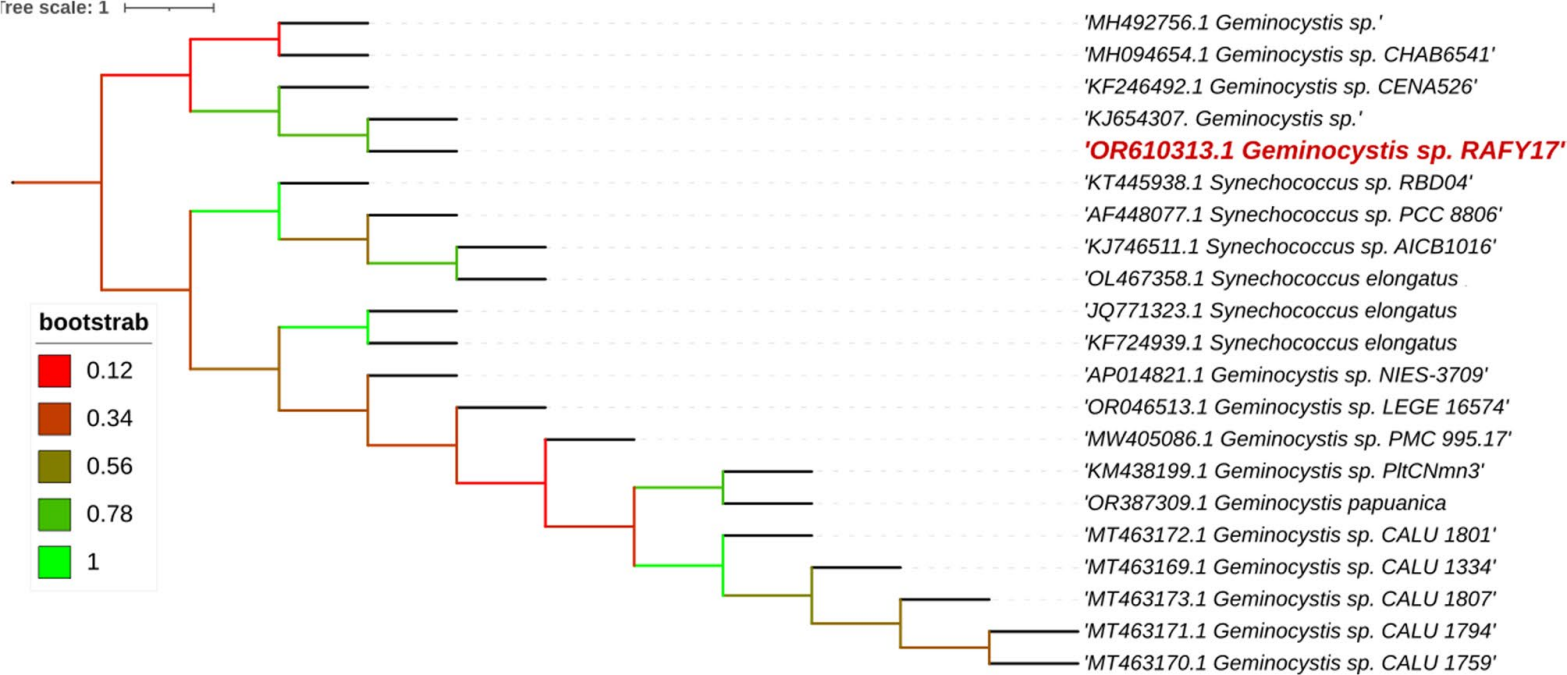
Phylogenetic tree of partial 16s rRNA showing the phylogenetic position of *Geminocystis *sp*. *(RAFY17)(written in red) compared with other related cyanobacteria from the Gene Bank. Branches were colored according to bootstrap values

Phylogenetic analysis of *Geminocystis* sp. and *Alkalinema pantanalense*, the evolutionary relationship of the cyanobacterial isolate *Geminocystis sp*. (RAFY17) is presented in Fig. 1, based on a phylogenetic tree constructed using 21 closely related sequences retrieved from GenBank. The isolate *Geminocystis* sp. RAFY17 (GenBank accession no. OR610313) was found to be most closely related to *Geminocystis* sp. (accession no. KJ654307.1), indicating a shared recent common ancestor. This relationship is supported by a bootstrap value of 78%, affirming a moderately strong level of phylogenetic confidence. The resulting clade is distinct from *Synechococcus*, reinforcing the generic placement of the isolate within the *Geminocystis* lineage.

**Fig. 2:**
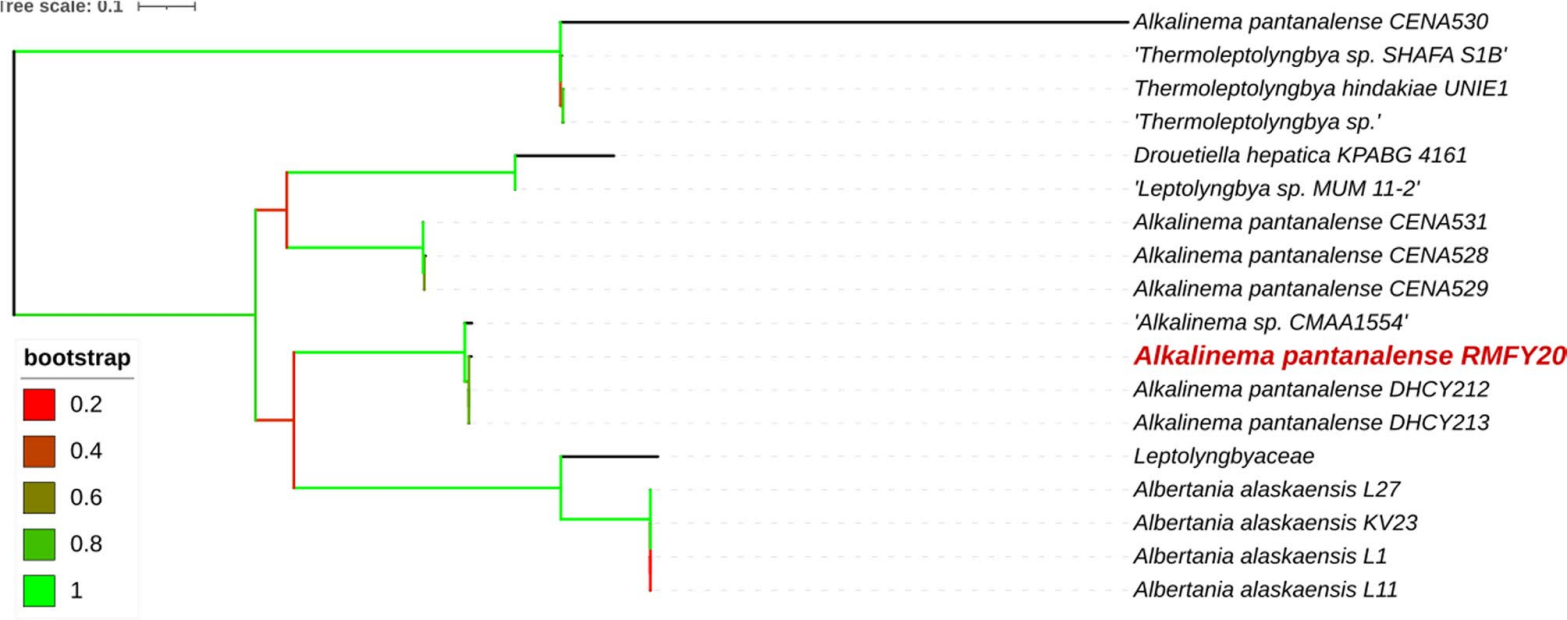
Phylogenetic tree of partial 16s rRNA showing the phylogenetic position of *Alkalinema pantanalense *(RMFY20)(written in red) compared with other related cyanobacteria from the Gene Bank. Branches were colored according to bootstrap values

Similarly, the phylogenetic position of *Alkalinema pantanalense* (RMFY20) is illustrated in Fig. 2. The tree was constructed using sequences from 18 algal taxa and resolved into three major clades. The isolate RMFY20 (GenBank accession no. OR469049.1) clustered closely with other *A. pantanalense* strains, including CENA531, CENA528, and CENA529, demonstrating a high degree of sequence similarity and evolutionary proximity. Furthermore, RMFY20 grouped with *A. pantanalense* isolates DHCY212 and DHCY213 within the same clade, supported by a bootstrap value of 100%, indicating robust phylogenetic confidence and confirming its taxonomic placement.

All phylogenetic trees were generated using the maximum likelihood method with 1,000 bootstrap replicates, providing strong statistical support for the inferred relationships and enhancing the reliability of the taxonomic classifications.

### Phytohormones, phenolic and flavonoid compounds

Each algal extract’s phytohormone, phenolic and flavonoid content were measured; the findings are shown in Table 2. All known phytohormones, except GBA, are more abundant in *Geminocystis* sp. than in *A.* *pantanalense*. The chromatogram of the phytohormones peaks is shown in Figure S3. Compared to *Geminocystis* sp.* A. pantanalense* has two additional phenolic compounds: caffeine and ferulic acid. Two flavonoid compounds (Quersestin and Luteolin) were seen in *A. pantanalense* and were not seen in *Geminocystis* sp., Also two others (Apegenin and Catechin) were seen in *Geminocystis* sp. and not found in *A. pantanalense*.

**Table 2:** Quantification of phenolic, flavonoid, and phytohormonal compounds in algal extracts (expressed as µg/g dry weight)

	*A. pantanalense*			*Geminocystis* sp.		
	Phenolic content					
	Area mAU*min		**Amount µg/g dw**	Area mAU*min		**Amount µg/g dw**
Chlorogenic	10.532		11.8156	15.935		33.3408
Catechol	2.292		2.5715	3.819		7.9912
Syringenic	0.994		1.1146	0.246		0.5146
p-coumaric	1.877		2.1058	0.250		0.5235
Cinnamic	17.842		20.0162	1.897		3.9687
Caffeic	4.254		4.7727	-		-
Ferulic	4.685		5.2556	-		-
	**Flavonoid compounds**					
Naringin	1.478	130.6752		0.823	68.8198	
Rutin	0.218	19.2420		1.178	21.5069	
Myricetin	0.166	14.7172		1.582	31.3045	
Quersestin	0.217	19.1671		**-**	**-**	
Luteolin	0.102	9.0232		**-**	**-**	
Apegenin	**-**	**-**		5.362	13.0874	
Catechin	**-**	**-**		6.403	23.6448	
	**Plant hormones**					
GBA	500.65	19.74		497.89	18.96	
6-Benzyle adenine	428.76	14.85		490.47	17.85	
IAA	480.65	16.85		502.65	20.95	
ABA	344.08	10.66		366.07	14.74	

Note: All values are expressed in micrograms per gram dry weight (µg/g dw) of algal biomass. The values were derived from HPLC quantification performed on ethanolic extracts prepared from 1 g dry algal biomass, as described in the Materials and Methods section

### GC-MS analysis of algae extracts

By the GC-MS analysis of *A. pantanalense* extract, 26 compounds were identified. The identification of compounds is based on the peak area and molecular weight. The result shown in Table 3 and Figure S3 indicate three prominent compounds, Olic acid (C_18_H_34_O_2_) with retention time of 30.53 and peak area of 20.99, à-Amyrin (C_30_H_50_O) with a retention time of 36.11 and peak area of 15.32 and Lupeol (C_30_H_50_O) with a retention time of 36.62 and peak area of 18.34. for *Geminocystis* sp. 20 compounds were reported (see Table 3; Fig. S4 ), where three prominent compounds were monitored; 9-Hexadecenoic acid, methyl ester, (Z)- (C_17_H_32_O_2_) with retention time of 25.91 and peak area 22.55, 8-Heptadecene (C_17_H_34_) with retention time of 21.91 and peak area of 11.70 and Phytol (C_20_H_40_O) with retention time of 29.93 and peak area of 14.29.

**Table 3:** GC-MS of *A. pantanalense* extract and *Geminocystis* sp.

A. pantanalense								
RT	Compound Name		Area %		Molecular Formula		Molecular Weight	
17.45	Phenol, 3,5-bis(1,1-dimethylethyl)-		0.42		C14H22O		206	
22.12	1-Hexadecanol, 2-methyl-		0.30		C17H36O		256	
24.03	1-Dodecanol, 3,7,11-trimethyl-		0.38		C15H32O		228	
24.69	1,2-Benzenedicarboxylic acid, butyl octyl ester		0.56		C20H30O4		334	
24.90	Ethanol, 2-(9-octadecenyloxy)-, (Z)-		1.04		C20H40O2		312	
25.09	1-Dodecanol, 3,7,11-trimethyl-		0.32		C15H32O		228	
25.37	Ethanol, 2-(9,12-octadecadienyloxy)-, (Z, Z)-		0.17		C20H38O2		310	
25.74	Z, E-2,13-Octadecadien-1-ol		0.25		C18H34O		266	
26.22	7,9,11-TRIMETHYL-3-PHENYL-2-OXO-4,6-DIAZATRICYCLO[5.3.2.0(1,5)]DODECA-3,5,8,11-TETRAENE-10-ONE		0.56		C18H16N2O2		292	
26.46	Hexadecanoic acid, methyl ester		1.33		C17H34O2		270	
27.41	n-Hexadecanoic acid		8.53		C16H32O2		256	
29.44	2-Methyl-Z, Z-3,13-octadecadienol		3.31		C19H36O		280	
29.61	10-Octadecenoic acid, methyl ester		1.82		C19H36O2		296	
29.93	Phytol		1.70		C20H40O		296	
30.16	Heptadecanoic acid, 16-methyl-, methyl ester		0.75		C19H38O2		298	
30.53	Oleic Acid		20.99		C18H34O2		282	
31.35	Corymbolone		1.38		C15H24O2		236	
31.55	trans-13-Octadecenoic acid		1.56		C18H34O2		282	
33.58	Ethyl iso-allocholate		0.40		C26H44O5		436	
36.11	à-Amyrin		15.32		C30H50O		426	
36.62	Lupeol		18.34		C30H50O		426	
38.07	Tricyclo[20.8.0.0(7,16)]triacontane, 1(22),7(16)-diepoxy-		0.15		C30H52O2		444	
39.98	LUP-20(29)-ENE-3,28-DIOL, (3á)-		9.61		C30H50O2		442	
41.12	Betulin		4.65		C30H50O2		442	
41.35	.psi.,.psi.-Carotene, 1,1’,2,2’-tetrahydro-1,1’-dimethoxy-		2.18		C42H64O2		600	
40.73		LUP-20(29)-ENE-3,28-DIOL, (3á)-		3.83		C30H50O2		442
42.33	ARABINITOL, PENTAACETATE		3.63		C15H22O10		362	
*Geminocystis* sp.								
RT	Compound Name		Area %		Molecular Formula		Molecular Weight	
17.44	1,4-BENZENEDIOL, 2-(1,1-DIMETHYLETHYL) −5-(2-PROPENYL)-		0.72		C13H18O2		206	
21.91	8-Heptadecene		11.70		C17H34		238	
22.36	Tridecanoic acid, 12-methyl-, methyl ester		10.58		C15H30O2		242	
23.59	Tetradecanoic acid		3.46		C14H28O2		228	
23.98	9-OCTADECENOIC ACID		1.07		C18H34O2		282	
24.68	Phthalic acid, butyl undecyl ester		0.49		C23H36O4		376	
24.89	9,12-Octadecadienoic acid (Z, Z)-		1.28		C18H32O2		280	
25.91	9-Hexadecenoic acid, methyl ester, (Z)-		22.55		C17H32O2		268	
26.40	Hexadecanoic acid, methyl ester		7.90		C17H34O2		270	
27.05	Palmitoleic acid		3.22		C16H30O2		254	
27.39	OleicAcid		4.32		C18H34O2		282	
29.43	12-Methyl-E, E-2,13-octadecadien-1-ol		1.35		C19H36O		280	
29.60	10-Octadecenoic acid, methyl ester		1.64		C19H36O2		296	
29.93	Phytol		14.29		C20H40O		296	
30.15	Heptadecanoic acid, 16-methyl-, methyl ester		3.24		C19H38O2		298	
30.81	cis-13-Octadecenoic acid		1.20		C18H34O2		282	
30.94	trans-13-Octadecenoic acid		0.65		C18H34O2		282	
31.61	cis-13-Eicosenoic acid		1.42		C20H38O2		310	
36.62	Bis(2-ethylhexyl) phthalate		4.63		C24H38O4		390	

### Growth parameters of wheat

**Fig. 3:**
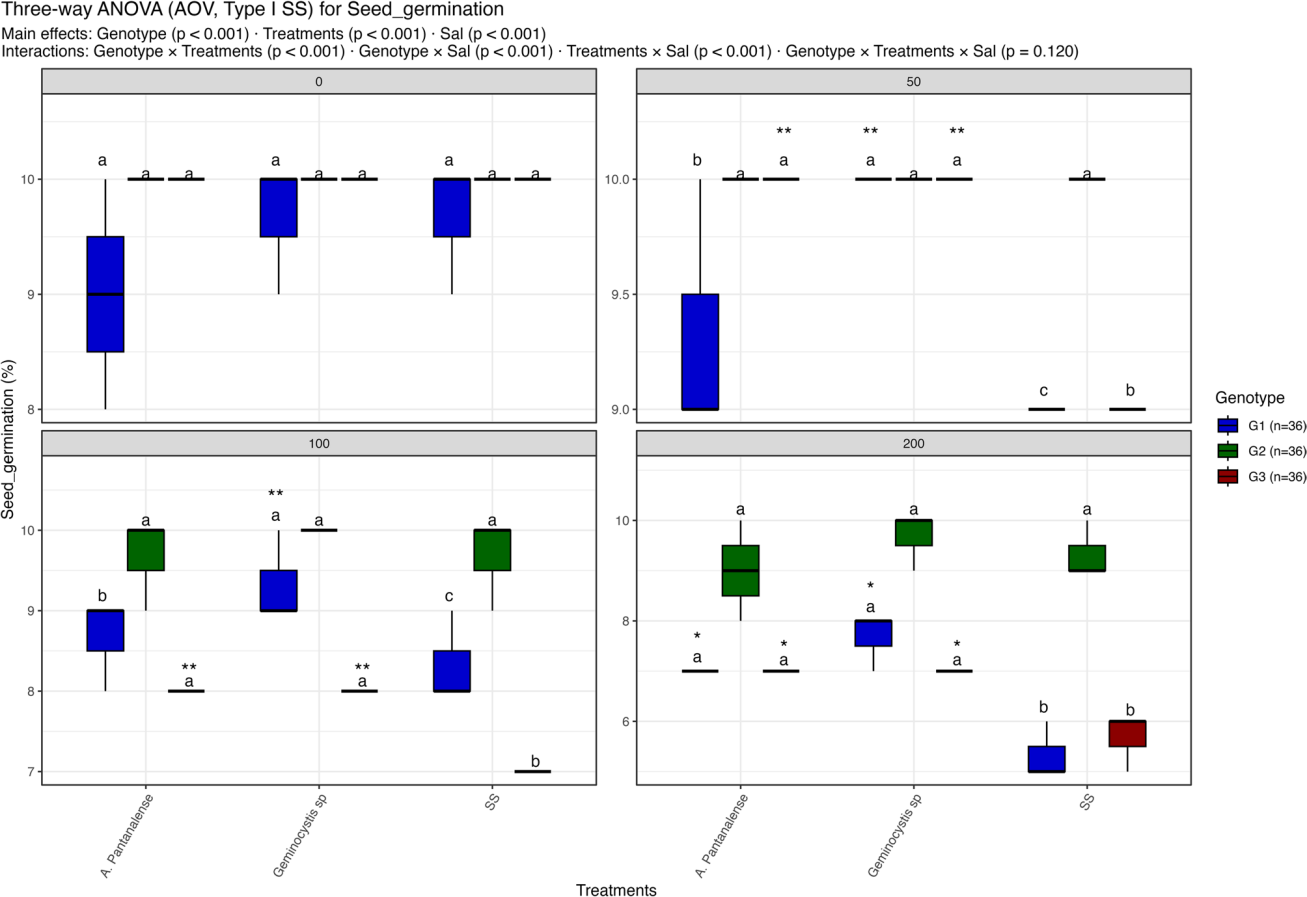
Distribution of seed germination percent under different treatment conditions: salt stress alone (“SS”), and salt stress combined with algal extracts from *A. pantanalense* or *Geminocystis* sp.. The data are presented across three wheat genotypes (G1, G2, and G3), represented by blue, green, and red boxplots, respectively. Data are presented as mean ± SE (*n* = 3). Statistical analyses were performed using three-way ANOVA followed by Tukey HSD for multiple comparisons. Different letters above the bars indicate significant differences among treatments at *p *< 0.05 within each genotype. stars indicate significance when compared to control (SS) within each genotype

**Fig. 4:**
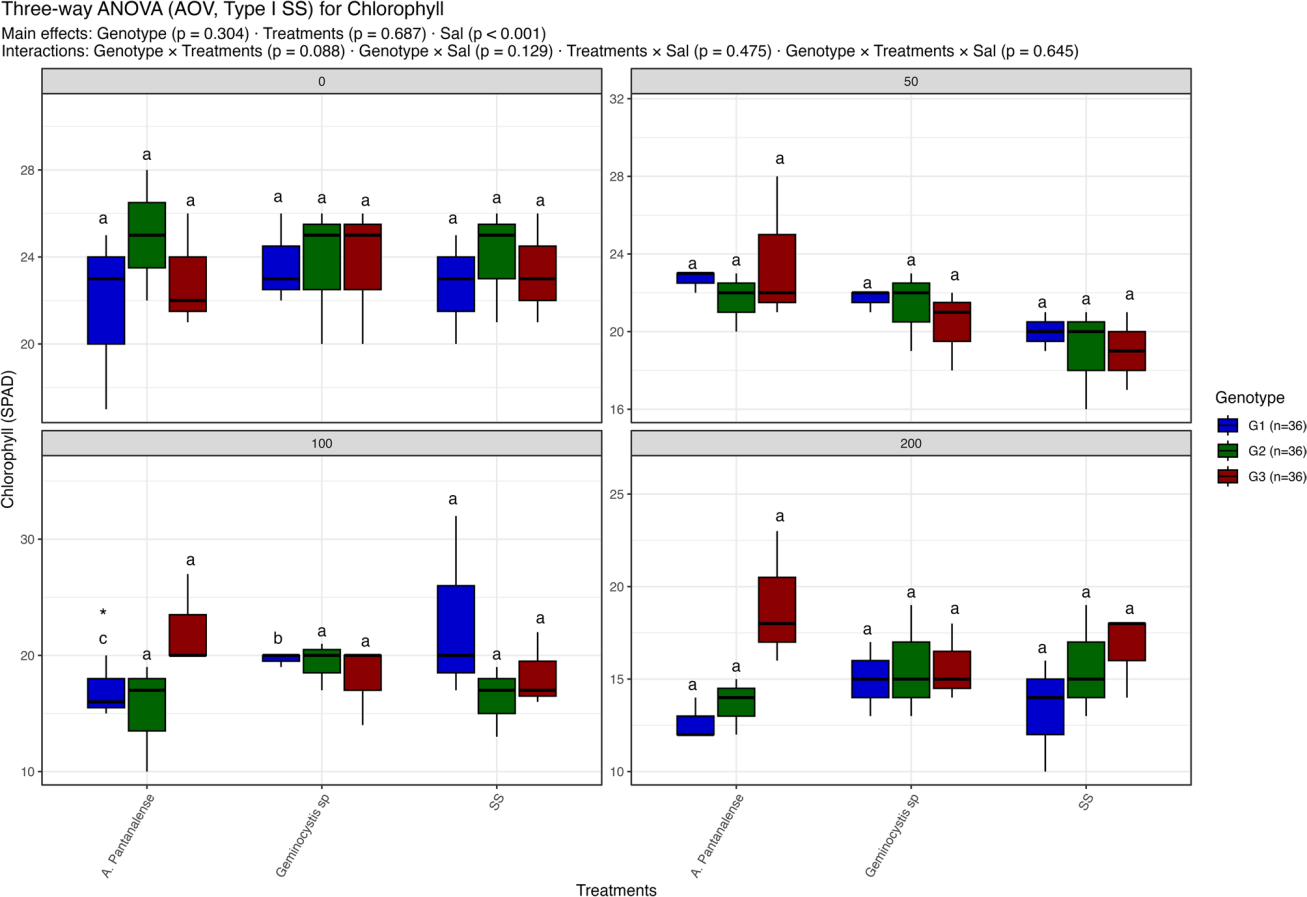
Distribution of chlorophyll under different treatment conditions: salt stress alone (“SS”), and salt stress combined with algal extracts from *A. pantanalense* or *Geminocystis* sp.. The data are presented across three wheat genotypes (G1, G2, and G3), represented by blue, green, and red boxplots, respectively. Data are presented as mean ± SE (*n* = 3). Statistical analyses were performed using three-way ANOVA followed by Tukey HSD for multiple comparisons. Different letters above the bars indicate significant differences among treatments at *p* < 0.05 within each genotype. stars indicate significance when compared to control (SS) within each genotype

**Fig. 5:**
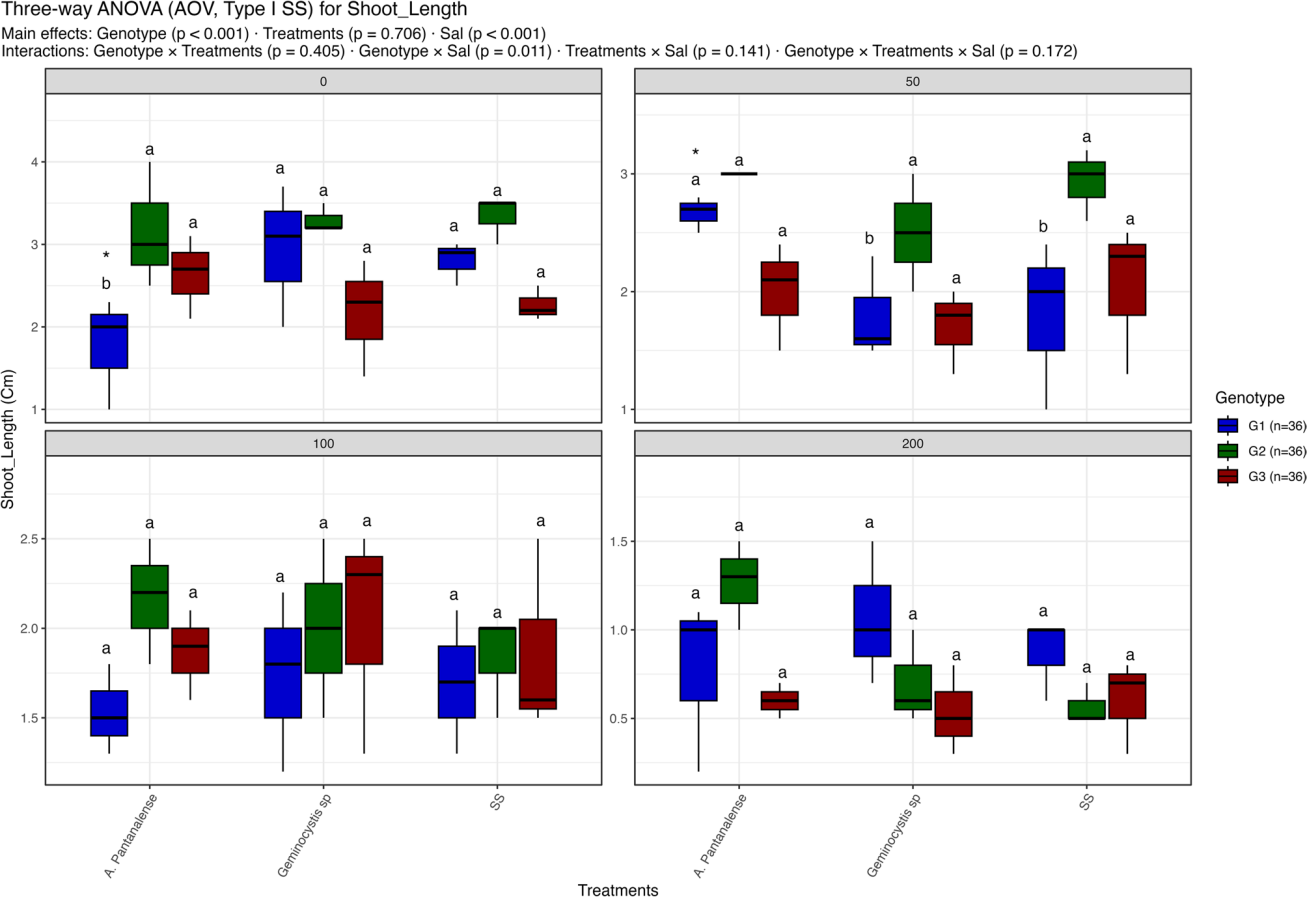
Distribution of shoot length under different treatment conditions: salt stress alone (“SS”), and salt stress combined with algal extracts from *A. pantanalense* or *Geminocystis* sp.. The data are presented across three wheat genotypes (G1, G2, and G3), represented by blue, green, and red boxplots, respectively. Data are presented as mean ± SE (*n* = 3). Statistical analyses were performed using three-way ANOVA followed by Tukey HSD for multiple comparisons. Different letters above the bars indicate significant differences among treatments at *p* < 0.05 within each genotype. stars indicate significance when compared to control (SS) within each genotype

**Fig. 6:**
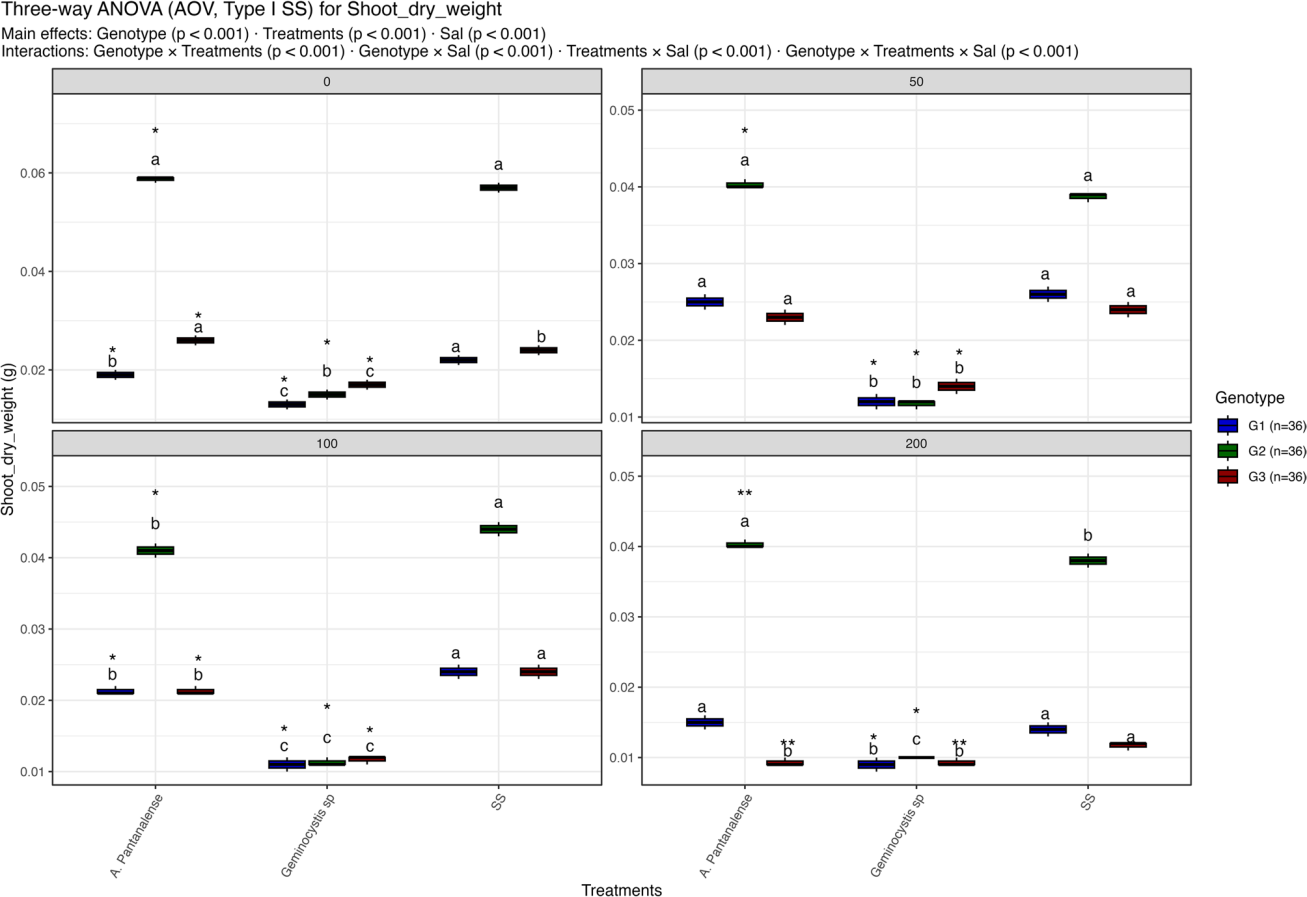
Distribution of shoot dry weight under different treatment conditions: salt stress alone (“SS”), and salt stress combined with algal extracts from *A. pantanalense* or *Geminocystis* sp.. The data are presented across three wheat genotypes (G1, G2, and G3), represented by blue, green, and red boxplots, respectively. Data are presented as mean ± SE (*n* = 3). Statistical analyses were performed using three-way ANOVA followed by Tukey HSD for multiple comparisons. Different letters above the bars indicate significant differences among treatments at *p* < 0.05 within each genotype. stars indicate significance when compared to control (SS) within each genotype

**Fig. 7:**
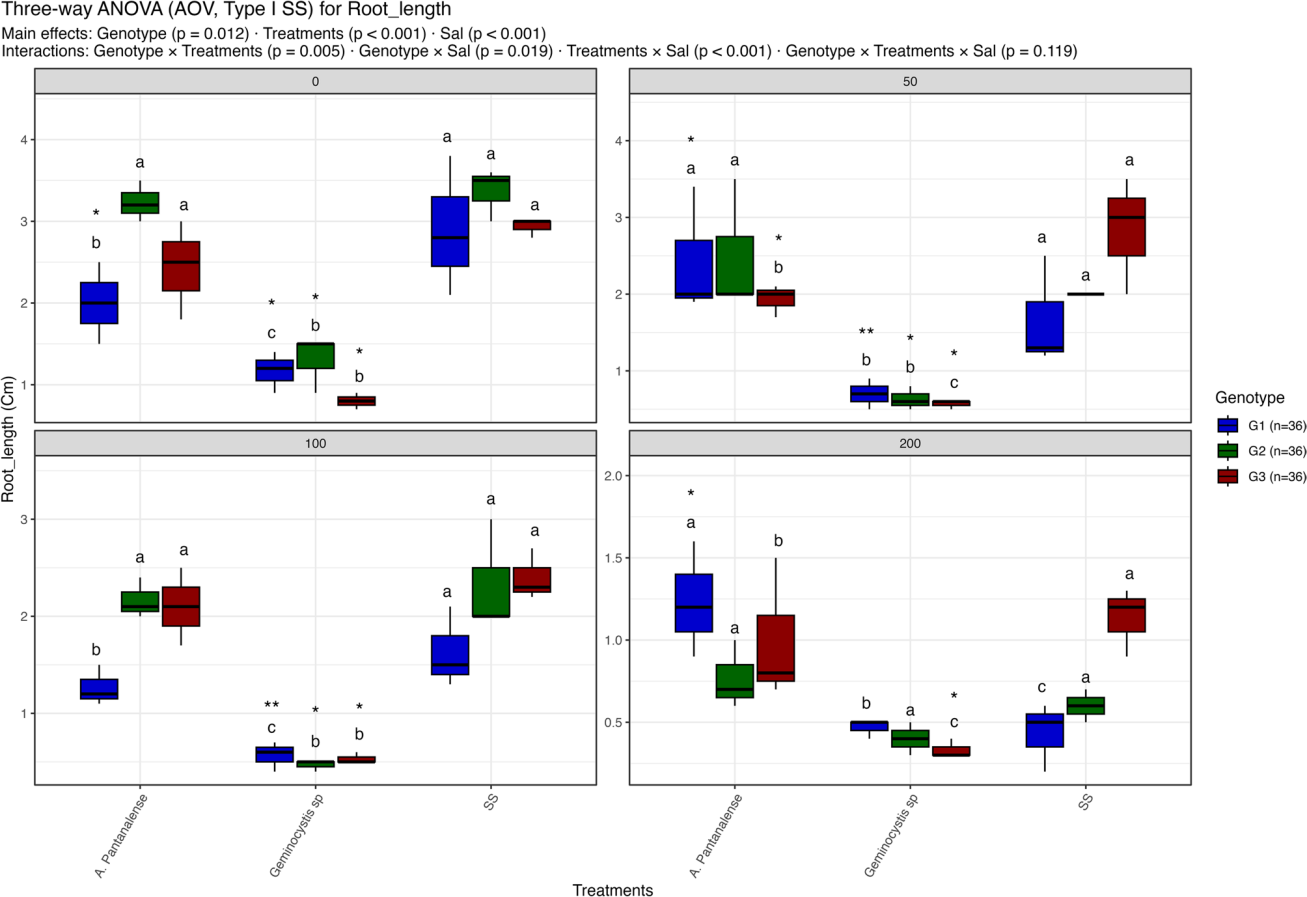
Distribution of root length under different treatment conditions: salt stress alone (“SS”), and salt stress combined with algal extracts from *A. pantanalense* or *Geminocystis* sp.. The data are presented across three wheat genotypes (G1, G2, and G3), represented by blue, green, and red boxplots, respectively. Data are presented as mean ± SE (*n* = 3). Statistical analyses were performed using three-way ANOVA followed by Tukey HSD for multiple comparisons. Different letters above the bars indicate significant differences among treatments at *p* < 0.05 within each genotype. stars indicate significance when compared to control (SS) within each genotype

**Fig. 8:**
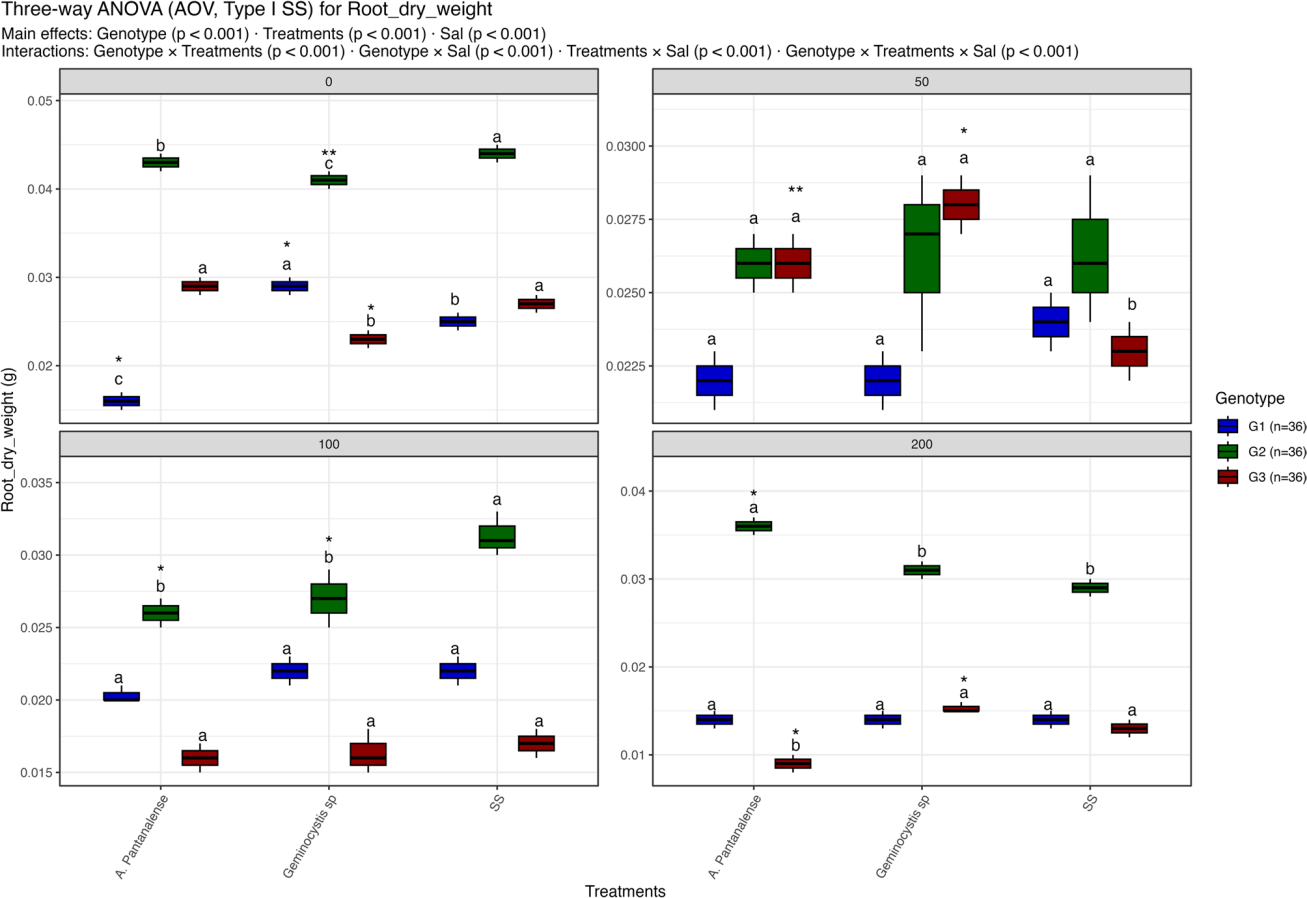
Distribution of root dry weight under different treatment conditions: salt stress alone (“SS”), and salt stress combined with algal extracts from *A. pantanalense* or *Geminocystis* sp.. The data are presented across three wheat genotypes (G1, G2, and G3), represented by blue, green, and red boxplots, respectively. Data are presented as mean ± SE (*n* = 3). Statistical analyses were performed using three-way ANOVA followed by Tukey HSD for multiple comparisons. Different letters above the bars indicate significant differences among treatments at *p* < 0.05 within each genotype. stars indicate significance when compared to control (SS) within each genotype

**Fig. 9:**
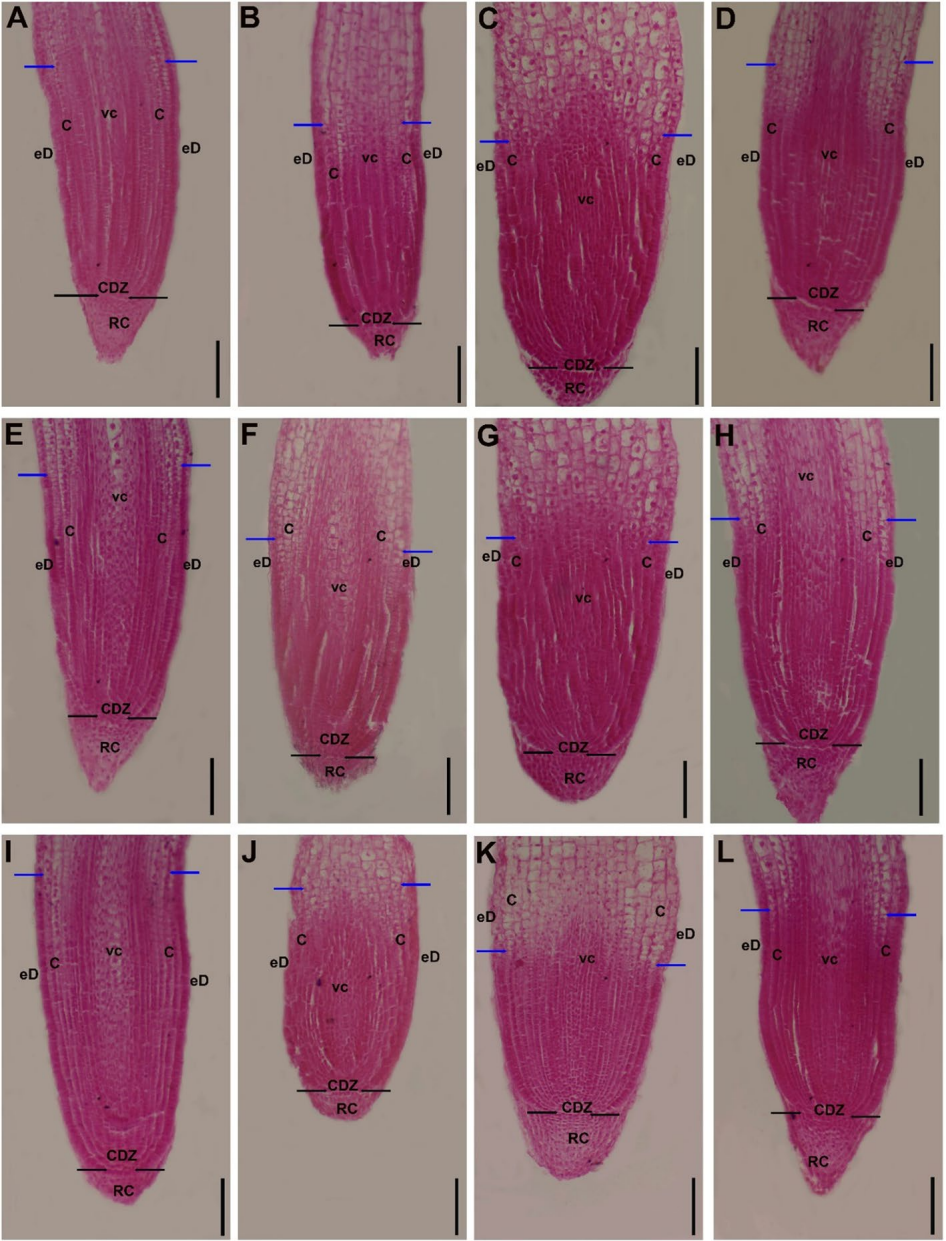
Longitudinal section (10μm) of 2 mm root tip of wheat (Genotypes1, 2, and 3; G1, G2, and G3, respectively) stained with saffranin showing structure of root tip zones. Root apical meristem of wheat seeds treated with water (control), 200mM NaCl solution, *Geminocystis* sp. treatment, or *A. pantanalense* treatment. A; G1-control, B; G1-200mM NaCl stress (SS), C; G1-200mM NaCl stress (SS) + *Geminocystis* sp. treatment, D; G1-200mM NaCl stress (SS) + *A. pantanalense* treatment, E; G2-control, F; G2-200mM NaCl stress (SS), G; G2-200mM NaCl stress (SS) + *Geminocystis* sp. treatment, H; G2-200mM NaCl stress (SS) + *A. pantanalense* treatment, I; G3-control, J; G3-200mM NaCl stress (SS), K; G3-200mM NaCl stress (SS) + *Geminocystis* sp. treatment, L; G3-200mM NaCl stress (SS) + *A. pantanalense* treatment. C; cortex, eD; epidermis, VC; vascular cylinder, CDZ; Cell division zone, RC; Root cap. Black arrows represent the quiescent center (QC), while blue arrows represent the transition zone (TZ). Bars represent 200μm

Figures 3, 4, 5, 6, 7, 8 and 9 present the effects of four different salt concentrations (0, 50, 100, 200 mM) single or in combination with two different algal strains (*A. pantanalense *and *Geminocystis* sp.) on six parameters (seed germination, chlorophyll content, shoot length, shoot dry weight, root length and root dry weight) across three distinct genotypes (G1, G2, and G3), represented by red, green, and blue boxplots, respectively Each treatment–genotype combination is labeled with significance groupings (a–e) derived from a post hoc comparison “Tukey HSD test” where shared letters denote no statistically, significant difference at *p* < 0.05.

#### Percent of seed germination

Figure 3 shows that genotype, treatment, and salinity level all had a substantial impact on seed germination, as did their interactions (three-way ANOVA; *p* < 0.001 for main effects and most interactions). There were no discernible changes between treatments, and all maintained consistently high germination rates (~ 9–10%) at 0 mM NaCl. The germination of treated groups remained reasonably high at 50 mM NaCl, but the control (SS) and some genotypes exhibited substantial decreases (*p* < 0.05). At 100 mM NaCl, genotypic variation was visible: SS showed the lowest germination percentages (< 8%), whereas G2 maintained significantly higher germination than G1 and G3 throughout most treatments. All genotypes showed a dramatic drop in germination at 200 mM NaCl, with the SS control showing values below 7%. On the other hand, *A. pantanalense* and *Geminocystis* sp. treatments helped G2 cope with some of the effects of salinity stress while preserving germination rates above 9%.

Letters indicating homogenous groups varied with treatment × salinity combinations, as confirmed by Tukey HSD post-hoc comparisons. At greater salinity levels, treated seeds (algal extracts) typically clustered in superior groups (“a”), whereas untreated controls (SS) fell into inferior groups (“b” or “c”). This pattern shows that algal extracts considerably reduced the detrimental effects of salt on seed germination, with G2 showing a greater protective impact than G1 and G3.

#### Chlorophyll content

While genotype and treatment main effects were not significant, salinity had a substantial impact on chlorophyll content (SPAD values) (*p* < 0.001) (Fig. 4). All treatments and genotypes showed comparatively constant chlorophyll levels at 0 mM NaCl, with no discernible variations. Although variations across genotypes and treatments were not statistically significant, a modest decrease in chlorophyll content was noted across treatments at 50 mM NaCl.

Variation became more noticeable at 100 mM NaCl. Under *A. pantanalense*, G1 showed noticeably more chlorophyll in the SS control than G2, although *Geminocystis* sp. treatments did not clearly show any advantage. With no discernible variations across treatments, the chlorophyll content dropped significantly in all groups at 200 mM NaCl, reaching ~ 12–17 SPAD units. This suggests that algal extracts were unable to counteract the salinity-induced decrease at this concentration. Thus, salinity decreased chlorophyll content, with genotype and algal extracts contributing only slight, non-significant variation, that was supported by post-hoc comparisons (Tukey HSD), which showed that letters representing homogenous groups overlapped widely across treatments.

#### Shoot length

Salinity (*p* < 0.001) and genotype (*p* < 0.001) had a substantial impact on shoot length, while treatment had no effect (*p* = 0.706) (Fig. 5). varying genotypes behaved differently to varying salt levels, as evidenced by a significant genotype × salinity interaction (*p* = 0.011).

Shoot length varied between ~ 2 and 3.5 cm at 0 mM NaCl, with G2 exhibiting the highest values, particularly when treated with *A. pantanalense*, and G1 having noticeably shorter shoots (~ 2 cm). All genotypes showed a modest loss in shoot length at 50 mM NaCl, with G2 maintaining comparatively higher values (~ 3 cm) whereas G1 and G3 show severe reductions to ~ 1.6–2.3 cm.

With no discernible variations across treatments, additional reductions were seen in all groups (1.5–2.7 cm) at 100 mM NaCl, suggesting that algal extracts offered only a moderate level of protection. All genotypes experienced a significant decrease in shoot length to less than 1.5 cm at 200 mM NaCl, with G1 and G3 exhibiting the largest decreases. While genotypic variation was more noticeable at lower salinity levels, Tukey HSD grouping verified that treatment differences were negligible within each salinity level.

Overall, these findings suggest that shoot elongation was significantly hindered by salinity stress, with G2 exhibiting comparatively stronger tolerance at mild salinity (0–50 mM). However, algal extracts failed to consistently mitigate at higher stress levels.

#### Shoot dry weight

Salinity, treatment, and genotype all had a substantial impact on shoot dry weight, as did their interactions (all p < 0.001; Fig. 6). Among all treatments, G2 had the largest shoot dry weight (~ 0.06 g) at 0 mM NaCl, much outweighing G1 and G3. Higher biomass accumulation was maintained in G2 by both *A. pantanalense. * and *Geminocystis* sp. treatments, however G1 and G3 displayed significantly lower values (< 0.03 g).

G2 once more maintained a very high shoot dry weight (~ 0.04 g) at 50 mM NaCl, but G1 and G3 showed notable decreases (0.015–0.025 g). *A. pantanalense* consistently outperformed Geminocystis sp. and SS control in maintaining shoot biomass, as seen by their clustering in inferior groups (“b” and “c”). While both G1 and G3 under SS or Geminocystis sp. decreased below 0.02 g, G2 under A. pantanalensis treatment maintained a slightly greater dry weight (~ 0.04–0.05 g) at 100 mM NaCl. The tendency continued at 200 mM NaCl, with G1 and G3 across treatments remaining < 0.015 g whereas G2 under A. pantanalensis retained ~ 0.04 g, greatly surpassing all other combinations. G2 treated with *A. pantanalense* consistently belonged to the superior group (“a”) across salinity levels, whereas G1 and G3 were placed in lower groups (“b” or “c”), according to Tukey HSD post-hoc tests. With G2 exhibiting strong resistance to salt when supplemented by *A. pantanalense* while G1 and G3 were extremely vulnerable regardless of treatment, these results reveal that shoot biomass is highly genotype-dependent.

#### Root length

Genotype (*p* = 0.012), treatment (*p* < 0.001), and salinity (*p* < 0.001) all had a substantial impact on root length; significant interactions were found between genotype × treatment (*p* = 0.005) and genotype × salinity (*p* = 0.019) (Figuer. 7). Root length varied from around 0.6 to 3.5 cm at 0 mM NaCl. The roots of seeds treated with A. pantanalensis and SS were noticeably longer (~ 2–3.5 cm), while the roots of seeds treated with *Geminocystis* sp. were the shortest (< 1.0 cm), especially in G1 and G3. Root length decreased at 50 mM NaCl, but A. pantanalensis kept much longer roots in G1 and G2 (~ 2–3 cm. On the other hand, *Geminocystis* sp., which grouped in the inferior group (“c”), consistently produced the shortest root lengths (< 1.0 cm). Although A. pantanalensis and SS continued to maintain substantial root growth (~ 1.3–2.1 cm) in G2 and G3, while G1 roots remained short (~ 0.7 cm), further decreases were visible at 100 mM NaCl. All genotypes and treatments showed a significant reduction in root length (< 1.2 cm) at 200 mM NaCl. whereas *Geminocystis* sp. treatments suffered the most (< 0.7 cm). *Geminocystis* sp. was assigned to lower groups (“b” or “c”) across salinity levels, but *A. pantanalense* and SS consistently clustered in superior groups (“a”), according to Tukey HSD classifications. These results show that root growth was extremely sensitive to salinity; however, the adverse effects were somewhat lessened by *A. pantanalense* and SS, while *Geminocystis* sp. consistently exacerbated stress effects.

#### Root dry weight

Salinity, treatment, and genotype all had a significant impact on root dry weight; all interactions were highly significant (*p* < 0.001; Figure. 8). Among all treatments, G2 had the highest root biomass (~ 0.04–0.05 g) at 0 mM NaCl, greatly outperforming both G1 and G3. On the other hand, G1 clustered in the inferior group (“c”) and had the lowest root dry weight (~ 0.02 g), whilst G3 had intermediate values. All genotypes showed a modest loss in root biomass at 50 mM NaCl, clustering in superior groups (“a”). The effects of the treatments became more apparent at 100 mM NaCl. G1 and G3 had a further decline in biomass (~ 0.015–0.02 g), whereas G2 maintained a much greater biomass (~ 0.03–0.035 g) under *A. pantanalensis* or SS. All groups experienced more significant decreases (< 0.03 g) at 200 mM NaCl. However, G1 and G3 continued to be grouped in the lowest groups (“a”), although G2 once more maintained comparatively higher biomass (~ 0.03–0.04 g), especially under * A. pantanalense* and SS.

*A. pantanalense* and SS continuously increased root biomass retention in G2 despite increasing salinity, according to post-hoc Tukey comparisons, however G1 and G3 remained extremely sensitive irrespective of treatment.

### Longitudinal sections of wheat root tip

**Fig. 10:**
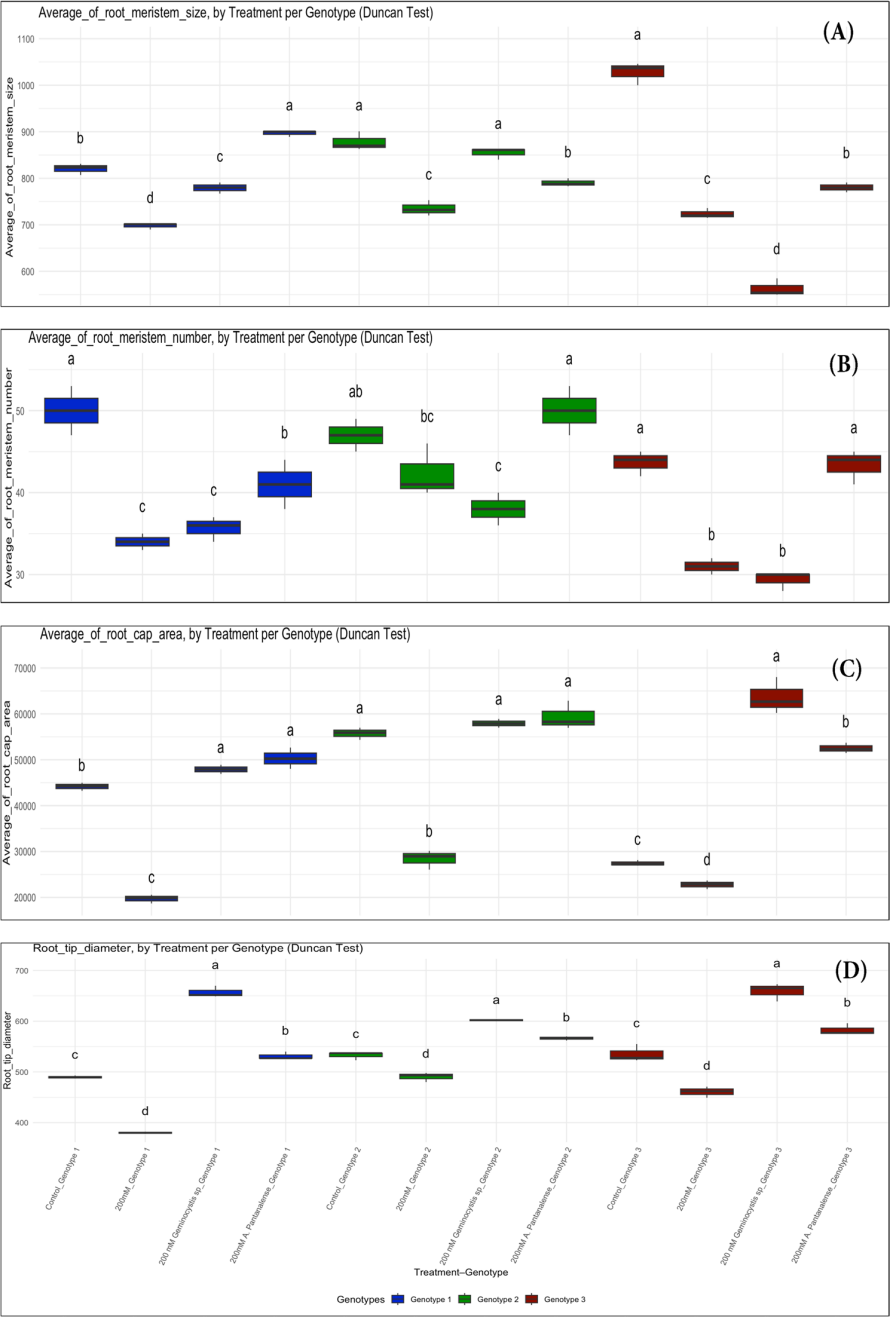
The anatomical parameters of the root apex (tip) of three wheat genotypes treated with *Geminocystis *sp. and green *A. pantanalense* algae under salt stress conditions at the seedling stage. A. Average of root meristematic size. B. Average of root meristem cell number. C. Average of root cap area. D. Average of root tip diameter. SS; salinity stress (200 mM NaCl stress). G1, G2, and G3 mean the genotype numbers. The represented data are values of means​​± standard deviations. Means labeled by different letters indicate significant differences between treatments according to Duncan HSD Multiple Range Test (*p* ≤ 0.05). Calyptrogen layer

A median longitudinal section of 10 μm in thickness and 2 mm in length, and stained with saffranin (Fig. 9), displayed the variation in the basic-closed organization of the root tip of wheat seedlings treated with two algae, i.e., brown (*Geminocystis* sp.) and green (*A. pantanalense*) algae, in addition to the control and 200 mM NaCl salt stress treatment (Fig. 10). The longitudinal sections of the root tip showed a significant difference (*p* < 0.05) in the average of root meristem size in the roots of the salinity-stressed wheat seedlings treated with *A. pantanalense* algae as compared with the salinity-stressed seedlings without algae treatment. Indicating that *A. pantanalense *algae have an important role in increasing the meristematic root tip cells that are responsible for the cell division mechanism in plant roots. Moreover, in G1 and G3, the salinity stressed wheat seedlings treated with *Geminocystis* sp. algae showed significantly (*p* < 0.05) lower values in the average of root meristem size as compared with roots tips of seedlings treated with *A. pantanalense* algae, indicating that the decrease of root length of the wheat seedling treated with *Geminocystis* sp. is related to its role in decreasing of root meristem size.

The average of root meristem cell number showed higher significant differences (*p* < 0.05) in the roots of the salinity stressed wheat seedlings treated with *A. pantanalense *algae as compared with the salinity stressed seedlings without algae treatment or seedling treated with *Geminocystis* sp. under sat stress, indicating that *A. pantanalense* algae participates in activation of root meristematic cell to promote root growth under salinity stress conditions.

The main function of the root cap area is to protect the root apical meristematic cell from damage by soil granules. The higher area of root cap means higher protective efficiency for root system especially under stress conditions, here, we reported that highly significant decrease (*p* < 0.05) in the root cap area due to salinity stress application as compared to the roots of control seedlings, while roots of wheat seedlings treated with *A. pantanalense* and *Geminocystis *sp. algae under salinity stress showed highly significant differences as compared with NaCl stressed seedlings without algae treatments.

Root tip diameter showed a significant difference in the roots of the salinity-stressed wheat seedlings treated with *A. pantanalense* and *Geminocystis* sp. algae as compared with control plants. Moreover, the highest increase in root tip diameter was reported in the roots of *Geminocystis* sp. algae-treated seedlings, indicating that *Geminocystis* sp. has an important role in improving the root volume of seedlings under salt stress conditions.

In general, our findings reported that the differentiation of the vascular cylinder (VC) showed well development in the control and *A. pantanalense* ensis algae-treated root tips, while the VC in the NaCl- and *Geminocystis *sp. algae-treated root tips under salt stress showed a weakness in the development (Fig. 9). In addition, the root tips treated with 200 mM sodium chloride or *Geminocystis* sp. algae under salt stress showed deep-stained blocks of relatively separated cells indicating abnormal cell division or elongation and loss of water from the cells contrast, in the control plants and *A. pantanalense* treated- root tips showed organized and normal cells (Fig. 9). In general, our findings reported that the *A. pantanalense* algae and salt treatments may reduce the division of cells of the Calyptrogen layer.

### Principal component analysis (PCA)

**Fig. 11:**
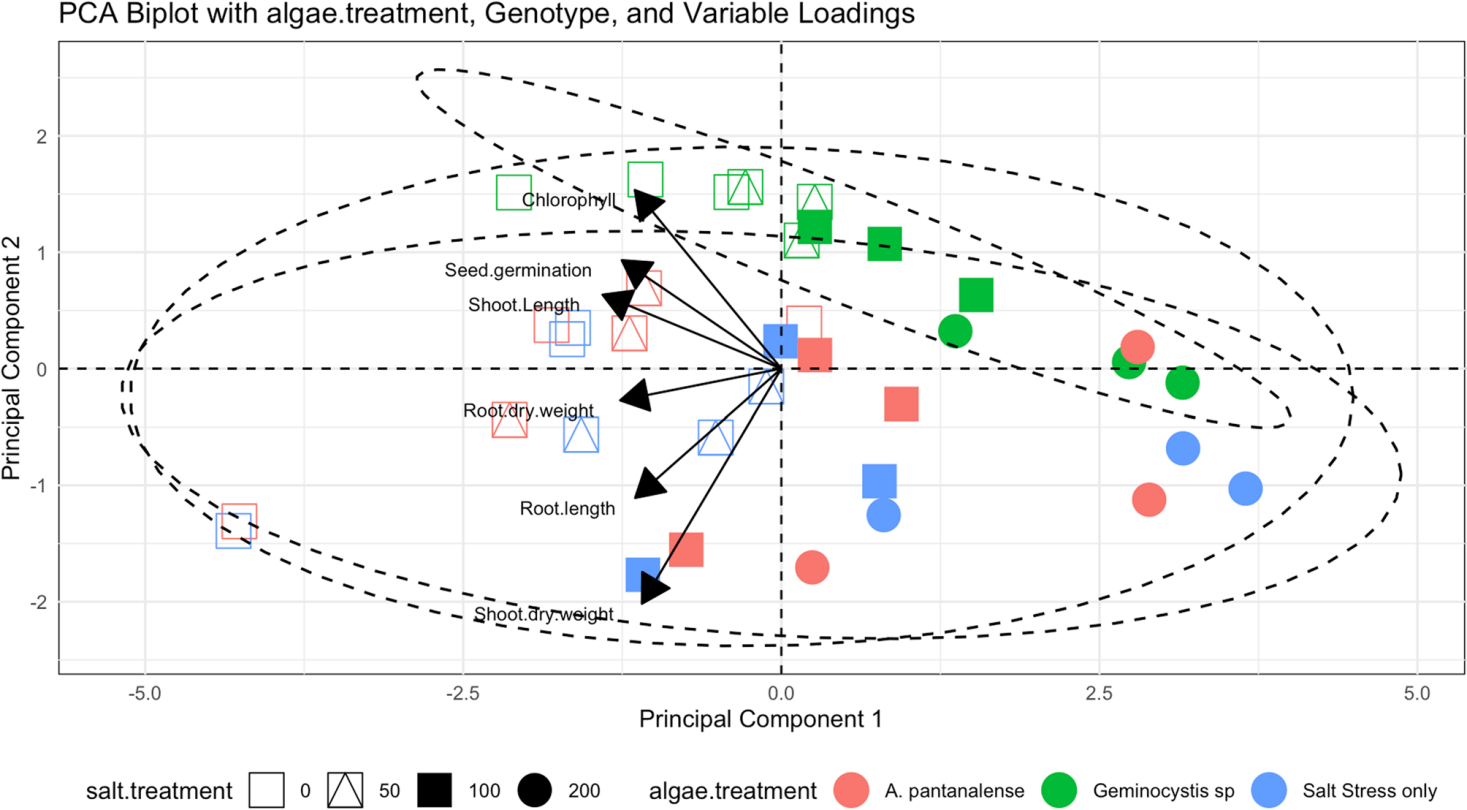
Principal component analysis (PCA) biplot illustrating the separation of all three genotypes in response to *Geminocystis *sp., based on measured growth parameters. Colored points represent different treatments, red *A. pantanalense*, green *Geminocystis *sp. and blue for salt stress only. while the shape reflects different salt treatment effects. Vectors indicate the contribution and direction of individual variables to the principal components

PCA was conducted on various wheat genotypes’ morphological and biochemical variables to illustrate the clustering and correlation among the analyzed genotypes under diverse treatments, including salinity, *A. pantanalense*, and *Geminocystis* extracts. The data presented in Figs. 11and S1 indicate that PCA1 explains 46.8% of the variation, but PCA2 only contributes 21.7%. The Principal Component Analysis (PCA) reveals a clear segregation of the genotypes treated with *Geminocystis* extract, forming a discrete cluster positioned in the upper quadrant of the plot. The factors of seed germination, shoot dry weight, and root dry weight are particularly prominent in this separation. The length of the arrow represents the magnitude of the variable, while the highest value of the variable is shown by the direction of the arrow. All genotypes exposed to salt stress and treated with *A. pantanalense* extract were classified in the lower quadrant, indicating their membership in overlapping groups.

### Heatmap correlation matrix

**Fig. 12:**
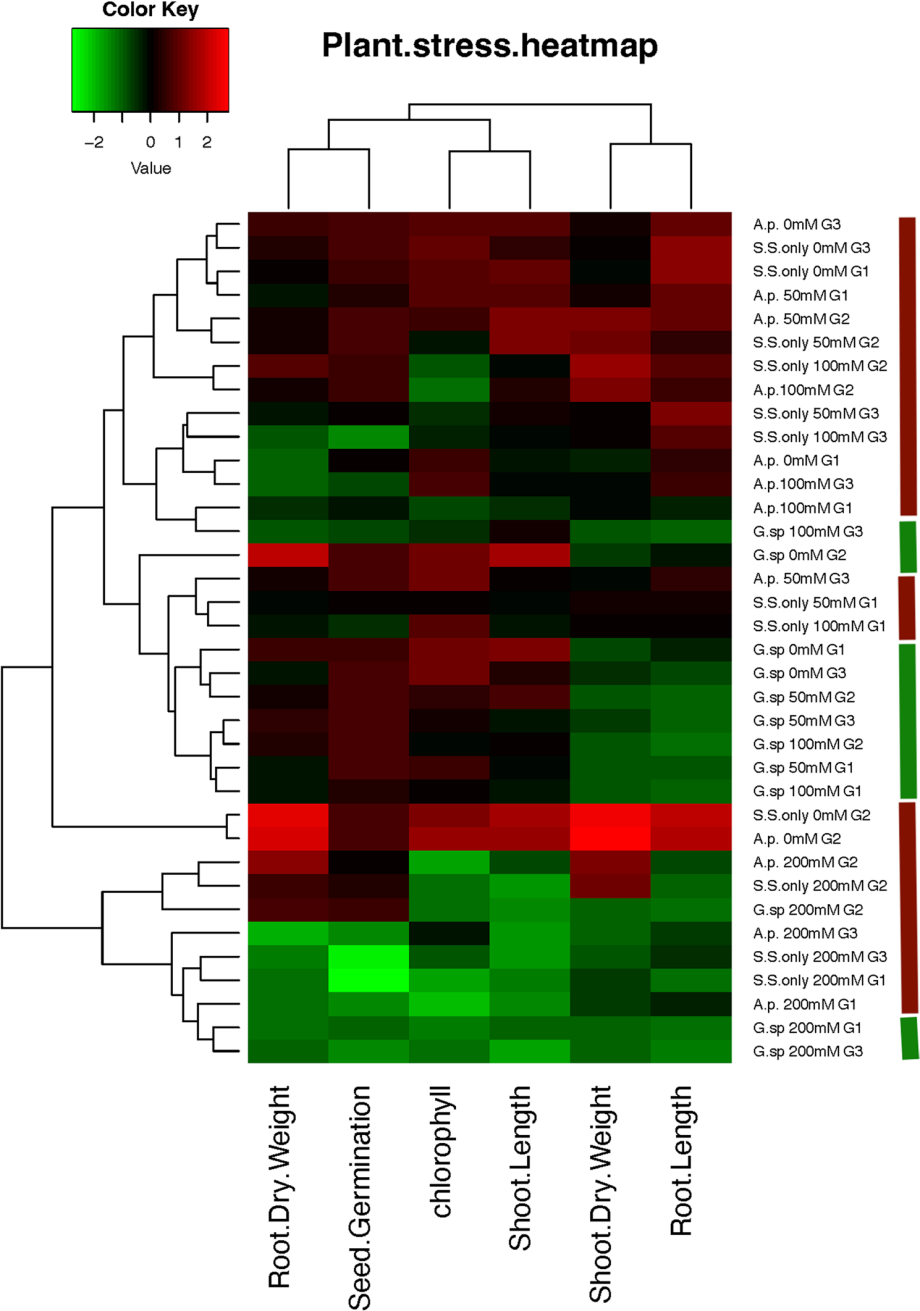
Correlation heatmap showing the relationships among four salt treatment (0, 50, 100 and 200)conditions (with and without algal extracts (G.sp is for *Geminocystis *sp , A.P. is for *A. pantanalense*, and ss is for salt stress only.)) and measured growth parameters across three wheat genotypes (G1, G2 and G3). The color scale indicates the strength and direction (positive or negative) of Pearson correlation coefficients

Heatmap Analysis of Plant Stress Parameters in Response to Salt Stress and Algal Treatments (Fig. 12). Figure 12 shows the hierarchical clustering of growth and physiological characteristics in plants treated with two algal species, *Geminocystis* sp. (G.sp.) and *A. pantanalense* (A.p.), and exposed to different salt concentrations. Six measured characteristics are included in the data: seed germination %, chlorophyll content, shoot length, root length, and root dry weight. Three genotypes (G1–G3) are included in the samples, which were treated either alone or in conjunction with algal supplementation under control and salt stress conditions (0–200 mM NaCl). With red denoting greater values and green denoting lower values in relation to the mean (z-score scale), colour intensity represents the relative performance of each feature. Strong treatment-dependent heterogeneity in plant responses is suggested by the clustering patterns seen along the rows and columns.

Notably, *Geminocystis *sp. treatments at moderate to high salt concentrations (50–200 mM) tend to group and exhibit generally higher values in root/shoot growth and chlorophyll content, indicating that this species may have a growth-promoting or protective effect under salinity stress. As seen by the noticeable green hues in those cells, salt-only treatments (S.S. only) without algal supplementation typically exhibit lower values, especially in seed germination and chlorophyll content.

Chlorophyll and seed germination are tightly related, which suggests that they co-regulate or drop in tandem under stress, according to hierarchical clustering of features. A discrete cluster of features relating to shoot and root biomass indicates that salinity and algal treatments have an impact on different physiological pathways. The conclusion that *Geminocystis *sp., especially in genotypes G2 and G3, has a stronger moderating impact on salt-induced stress than *A. pantanalense* is supported by this heatmap. These results support the possibility of using particular cyanobacteria as biostimulants in saline farming.

### Pearson correlation analysis of bioactive compounds vs. growth traits

**Fig. 13:**
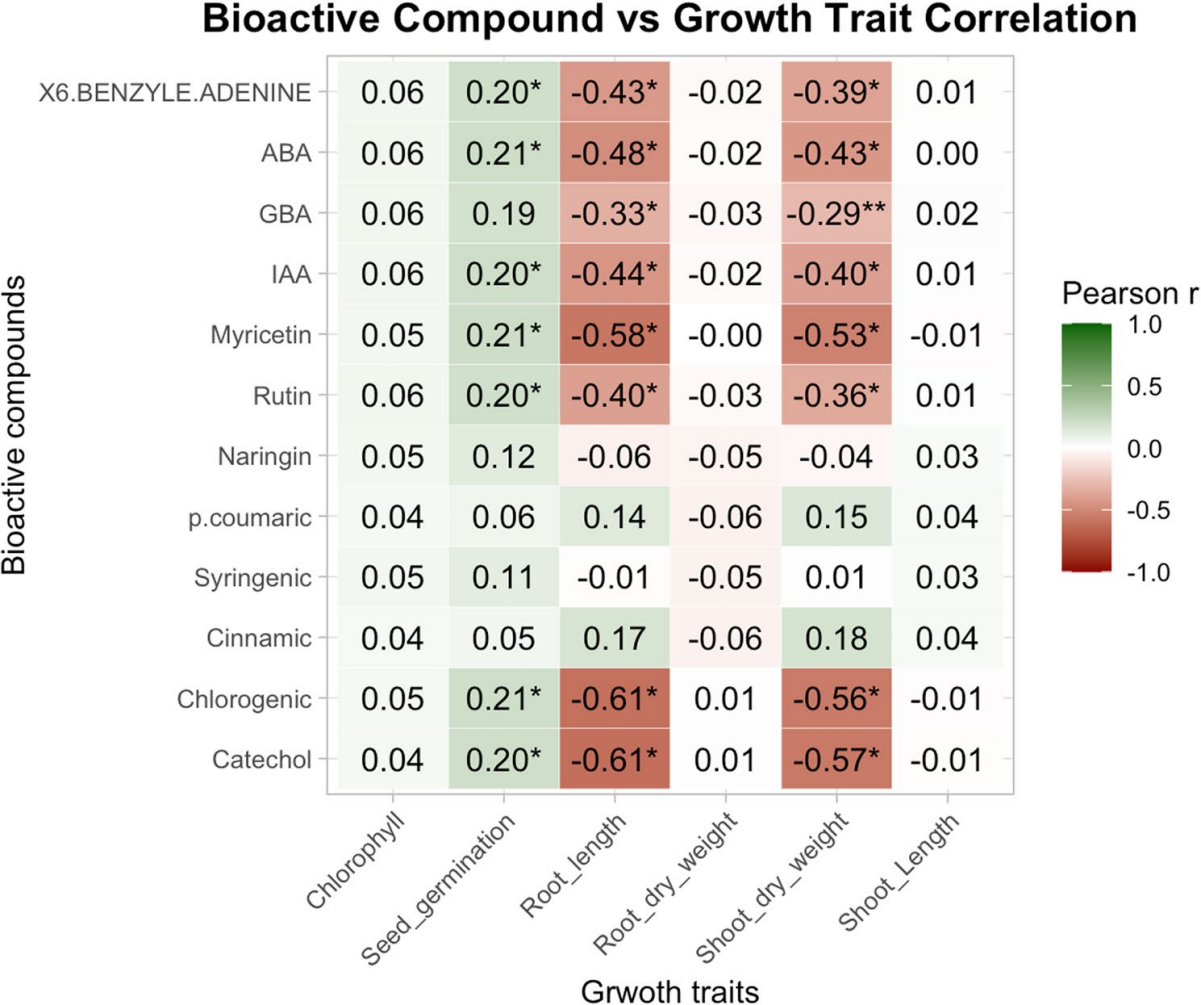
Heatmap of the Pearson association between wheat growth traits and the bioactive compounds. Individual bioactive substances (y-axis) and growth parameters (x-axis), are shown in the heatmap along with the direction and strength of the correlation (r). Positive correlations are represented by green hues, negative correlations by brown hues, and the strength of the correlation is shown by the intensity

A Pearson correlation heatmap was created in order to clarify the connections between the concentrations of bioactive compounds and growth-related metrics in treated wheat genotypes (Fig. 13). The correlation coefficients between six important growth traits—chlorophyll content, seed germination, root length, root dry weight, shoot dry weight, and shoot length—and 13 discovered bioactive substances are depicted in the heatmap along with their strength and direction.

Among the phytohormones, benzyled adenine, ABA, GBA, and IAA exhibited significant positive correlations with seed germination (*r* = 0.20–0.21; *p* < 0.05) but were negatively correlated with both root length and shoot dry weight. In particular, there were moderate negative correlations between ABA (*r* = − 0.48) and IAA (*r* = − 0.44) and root length (*p* < 0.05), as well as a negative association between GBA and IAA and shoot dry weight (*p* < 0.01). This implies that elevated hormone concentrations may have an inhibitory influence on biomass accumulation and root elongation.

Interestingly, the largest negative relationships with root and shoot parameters were seen for myricetin, catechol, and chlorogenic acid. Root length (*r* = −0.58) and shoot dry weight (*r* = −0.53) were significantly and negatively correlated with myricetin, whereas root length (*r* = −0.61) and shoot dry weight (*r* = −0.56 and − 0.57, respectively; *p* < 0.05) were consistently and significantly correlated negatively with chlorogenic acid and catechol. certain results might suggest that certain phenolic chemicals have phytotoxic effects or hinder the growth of roots and shoots under the investigated conditions.

On the other hand, the only substances that showed a positive association with shoot length were p-coumaric acid and cinnamic acid (*r* = 0.15 and 0.18, respectively), however these correlations were not statistically significant. All examined growth traits showed mild and largely non-significant associations with rutin, syringic acid, and naringin.

Overall, the correlation analysis emphasizes the complex physiological impacts of distinct bioactive chemicals in response to stress and treatment circumstances, highlighting both promotive and inhibitory relationships between these compounds and certain plant growth indices.

## Discussion

Salinity is a major abiotic stressor affecting nearly 20% of the world’s irrigated land and disrupt plant physiological functions, leading to reduced germination, stunted growth, and poor yield quality [57, 58]. As traditional chemical fertilizers increasingly burden ecosystems, sustainable alternatives such as biostimulants derived from microalgae have emerged as promising solutions. Microalgal extracts are rich in phytohormones, trace elements, amino acids, polysaccharides, and antioxidant biocompounds that confer tolerance to salt stress by modulating gene expression, hormone signaling, ion homeostasis, and antioxidative defenses [19, 59]. In this study, two cyanobacterial strains, *Geminocystis* sp. and *Alkalinema pantanalense*, were evaluated for their biostimulatory potential under salinity stress (0–200 mM NaCl) across three genotypes of Triticum aestivum (wheat). The measured parameters included seed germination percentage, chlorophyll content, shoot and root lengths, shoot and root dry weight, root anatomy, and bioactive compounds in the two algae, along with multivariate analyses (PCA and HCA), providing comprehensive insight into physiological resilience mediated by these microalgae. These findings are interpreted in light of relevant research on other crops treated with algal biostimulants, notably *Spirulina platensis* in wheat and *Chlorella* *vulgaris* and *Ettlia pseudoalveolaris* in quinoa, offering a broader scientific context for the observed phenomena [61]. Seed germination is the initial and most critical phase of plant development, particularly sensitive to salt stress, which hampers water uptake and disrupts metabolic activation by altering osmotic potential and hormonal signaling [62, 63]. In the current study, salinity caused a genotype-specific decline in germination, most pronounced in G1 and G3 at high NaCl concentrations (Fig. 3). However, treatment with *Geminocystis *sp. or *A. pantanalense *mitigated this reduction, particularly in G2, which maintained consistently high germination percentages across all treatments. This ameliorative effect can be attributed to bioactive constituents within the algal extracts. Previous studies report that algal-derived gibberellins and cytokinins stimulate the mobilization of storage reserves and cell division during early germination stages. For instance, *Spirulina platensis* extracts elevated wheat germination indices significantly, including the germination percentage (GP), seedling vigor index (SVI), and relative root elongation (RRE), by modulating hormonal balance and antioxidant capacity [67]. Moreover, microalgal phytohormone profiling in the present study revealed that *Geminocystis* sp. contained higher levels of indole-3-acetic acid (IAA), kinetin, and zeatin compared to *A. pantanalense*, supporting its superior performance in promoting germination under osmotic stress [68–70]. These hormones not only enhance cell division and elongation but also upregulate aquaporin activity, facilitating water uptake in salt-stressed seeds Field [71–73].

Chlorophyll is the cornerstone of photosynthesis and an early marker of stress-induced damage. Under salt stress, chlorophyll degradation often results from ROS accumulation and impaired magnesium uptake, both of which destabilize chloroplast membranes [74]. In this study, increasing salinity led to a marked decline in chlorophyll content, especially in genotype G1. However, algal treatments, particularly *A. pantanalense*, significantly ameliorated chlorophyll loss, with notable responses in G2 and G3 at 50–100 mM NaCl (Fig. 4). These observations align with findings in quinoa, where *Ettlia pseudoalveolaris* protected chlorophyll a and b content under salt stress by enhancing Mg²⁺ and Fe²⁺ uptake, both essential for chlorophyll biosynthesis [61]. While antioxidant effects are frequently associated with flavonoids and phenolic compounds, no direct antioxidant activity (e.g., ROS levels or enzymatic assays) was measured in this study. Therefore, any reference to ROS scavenging should be interpreted as a hypothesis based on the known biochemical properties of the compounds detected [75]. Flavonoids such as naringin, rutin, and myricetin, which were abundant in *A. pantanalense*, have been widely reported to scavenge reactive oxygen species and protect cellular membranes in plants exposed to abiotic stress (Agati et al. 2012). Similarly, phenolic acids like ferulic and caffeic acid may contribute to membrane stabilization and the regulation of oxidative responses. The improved chlorophyll retention and root meristem structure observed in our study may reflect such protective roles, although this requires experimental validation through the quantification of antioxidant enzymes (e.g., SOD, CAT) or non-enzymatic scavenging capacity in future studies [76, 78].

Roots are the primary interface for nutrient and water acquisition, and thus central to salinity adaptation. Salinity often restricts root growth through ionic toxicity, reduced hydraulic conductivity, and disruption of auxin gradients [79–85]. The increased diameter may reflect cellular thickening or radial growth as a structural response to osmotic pressure, albeit at the cost of longitudinal growth [86, 87]. Previous studies where seaweed and cyanobacterial biostimulants preserved cellular integrity and vascular architecture in stressed roots, partly due to enhanced antioxidant protection and hormone-like activity [75, 88]. Preservation of the calyptrogen layer and root cap area in *A. pantanalense*-treated roots may further buffer mechanical damage during growth in saline soil environments.

The biostimulant effects observed in this study can be mechanistically linked to the phytochemical profiles of *A. pantanalense* and *Geminocystis* sp., which were comprehensively characterized via flavonoid profiling, phenolic quantification, phytohormone content, and GC-MS analysis. These compounds have been shown to mitigate oxidative damage in roots by stabilizing membrane lipids and scavenging ROS generated under salt stress [89, 90]. The higher concentration of flavonols in *A. pantanalense* aligns with its observed effectiveness in maintaining meristem size and vascular organization. These phenolics are not only potent antioxidants but also play roles in lignin biosynthesis and cell wall reinforcement, contributing to anatomical resilience. *Geminocystis *sp., although possessing fewer phenolic species, was rich in chlorogenic acid, a compound with known antimicrobial and cytoprotective roles in stressed plants [91, 92].

The hormonal landscape of the two algal extracts further elucidates their distinct physiological effects. *Geminocystis* sp. contained higher levels of indole-3-acetic acid (IAA, 20.95 µg/g), 6-benzyl adenine (BA, 17.85 µg/g), and abscisic acid (ABA, 14.74 µg/g) compared to *A. pantanalense* (Figure S2). These profiles corroborate earlier studies where S. platensis extracts rich in auxins and cytokinins modulated shoot/root ratios and enhanced enzymatic responses in *Triticum aestivum* under stress [60]. The co-occurrence of elevated ABA in *Geminocystis* sp. may further counteract auxin-driven elongation, reinforcing an adaptive rather than growth-promoting response, favoring root thickening and osmotic adjustment [93–95]. These properties likely contribute to the maintenance of root architecture and vascular differentiation observed in *A. pantanalense*-treated plants, especially under 100–200 mM NaCl stress. The physiological benefits may thus stem from a synergy between moderate hormone content and membrane-protective secondary metabolites [96, 97].

Gas chromatography–mass spectrometry (GC-MS) of *A. pantanalense *revealed 26 distinct bioactive compounds, with prominent peaks for oleanolic acid (C_18_H_34_O_2_), lupeol (C_30_H_50_O), and α-amyrin, all known for their anti-inflammatory, antimicrobial, and cytoprotective properties (Figure S3 & S4). These compounds are known to enhance lipid metabolism and maintain membrane fluidity under salinity-induced oxidative stress [98–100]. The contrasting fatty acid profiles observed in the two cyanobacterial strains likely contributed to their differential effects on plant stress physiology. *A. pantanalense* exhibited a high content of oleic acid (C18:1), which comprised 20.99% of its extract composition. Therefore, oleic acid may play a role in preserving the root meristem and vascular cylinder organization observed in *A. pantanalense*-treated wheat roots [101, 104].

Hierarchical clustering (HCA) (Fig. 13) grouped treatments based on the co-regulation of traits such as chlorophyll content and germination, highlighting trait-specific responses to biostimulants and salt levels. These clustering patterns underscore the complexity of plant responses and the importance of holistic, data-driven evaluation when selecting biostimulants for specific crops or genotypes [105, 106].

Comparative evaluation with quinoa (*Chenopodium quinoa*) studies by Fiorentino et al. (2025) reveals both convergence and divergence [61]. While both microalgal extracts improved antioxidant defenses and pigment stability under salinity, varietal specificity was more pronounced in quinoa, suggesting greater genotype–biostimulant interaction in pseudocereals. Similarly, Spirulina-based research on wheat corroborates the growth-promoting and antioxidant-enhancing effects observed here, particularly under moderate salt conditions [60]. These parallels support the broader applicability of cyanobacterial biostimulants across plant taxa, while also emphasizing the need for dose-genotype matching.

Although the presence of antioxidant-rich metabolites was confirmed, the physiological relevance remains speculative, as ROS levels and oxidative stress biomarkers (e.g., MDA, electrolyte leakage) were not assessed in this study. Future research should directly measure these parameters to validate the proposed mechanisms.

## Conclusion

This study demonstrates the distinct biostimulant potential of two cyanobacteria—*Alkalinema pantanalense *and *Geminocystis* sp.—in mitigating salinity stress in wheat seedlings. Their effectiveness is linked to differences in phytohormone levels, phenolic and flavonoid composition, and fatty acid profiles. A. pantanalense, in particular, promoted chlorophyll retention, vascular development, and root meristem activity, while *Geminocystis *sp. improved root biomass and structural adaptation. These effects were genotype-dependent, underscoring the importance of matching biostimulant type to cultivar. From a practical standpoint, the use of algal extracts is promising for low-input, sustainable agriculture. Cyanobacteria are fast-growing, require minimal resources, and can be cultivated in non-arable environments using saline or wastewater. The water-based extracts used in this study are simple to prepare and could be formulated for seed priming, foliar spray, or irrigation in field settings. For farmers in salt-affected regions, such biostimulants may enhance early seedling establishment and stress resilience, especially when integrated with genotype selection and precision irrigation. Future research should focus on field-level validation, cost–benefit analysis, and optimization of extract concentration and application frequency. Additionally, pilot programs and participatory trials with local farmers could facilitate technology adoption and provide insights into operational scalability. In this way, marine and freshwater cyanobacteria may offer an affordable, eco-friendly alternative to synthetic growth enhancers in salinity-prone agroecosystems.

## Data Availability

All data generated or analysed during this study are included in this published article and its supplementary information files.

## Abbreviations

ROS: Reactive oxygen species
FAO: Food and Agriculture Organization
CDZ: Cell division zone
RC: Root cap
VC: Vascular cylinder

## References

[1] United Nations. World population prospects 2019: highlights. New York: United Nations, Department of Economic and Social Affairs, Population Division;; 2019.

[2] Elkelish A. New plant extracts toward multidrug resistance: the convergence of nanotechnology and nanoscience. Spectr Sci J. 2024;1:1–14. 10.3390/ijms252212368.

[3] Shrivastava P, Kumar R. Soil salinity: a serious environmental issue and plant growth promoting bacteria as one of the tools for its alleviation. Saudi J Biol Sci. 2015;22:123–31. 10.1016/j.sjbs.2014.12.001. 25737642 10.1016/j.sjbs.2014.12.001 PMC4336437

[4] Elkelish A, Abu-Elsaoud AMM. Crosstalk between abiotic and biotic stress responses in plants: mechanisms, outcomes, and implications for crop improvement. Spectr Sci J. 2024;1:27–34. 10.21608/sasj.2024.396598.

[5] Zhu JK. Abiotic stress signaling and responses in plants. Cell. 2016;167(2):313–24. 10.1016/j.cell.2016.08.029. 27716505 10.1016/j.cell.2016.08.029 PMC5104190

[6] FAO ITPS. Status of the World’s Soil Resources (SWSR) –Main Report. Rome, Italy: Food and Agriculture Organization of the United Nations and Intergovernmental Technical Panel on Soils; 2015.http://www.fao.org/3/a-i5199e.pdf

[7] Machado RMA, Serralheiro RP. Soil salinity: effect on vegetable crop growth. Management practices to prevent and mitigate soil salinization. Horticulturae. 2017;3:30. 10.3390/horticulturae3020030.

[8] Busoms S, Fischer S, Yant L. Chasing the mechanisms of ecologically adaptive salinity tolerance. Plant Commun. 2023;4:100571. 10.1016/j.xplc.2023.100571. 36883005 10.1016/j.xplc.2023.100571 PMC10721451

[9] Arif Y, Singh P, Siddiqui H, Bajguz A, Hayat S. Salinity induced physiological and biochemical changes in plants: an omic approach towards salt stress tolerance. Plant Physiol Biochem. 2020;156:64–77. 10.1016/j.plaphy.2020.08.042. 32906023 10.1016/j.plaphy.2020.08.042

[10] Rouphael Y, Colla G, editors. Biostimulants in Agriculture. Frontiers Media SA; 2020.10.3389/fpls.2020.00040 10.3389/fpls.2020.00040 PMC7010726 32117379

[11] Hachicha R, Elleuch F, Hlima B, Dubessay H, de Baynast P, Delattre H, Pierre C, Hachicha G, Abdelkafi R, Michaud S, P., Fendri I. Biomolecules from microalgae and cyanobacteria: applications and market survey. Appl Sci. 2022;12. 10.3390/app12041924.

[12] Nandagopal P, Steven AN, Chan L-W, Rahmat Z, Jamaluddin H, Mohd noh NI. Bioactive metabolites produced by cyanobacteria for growth adaptation and their pharmacological properties. Biology. 2021;10:1061. 10.3390/biology10101061. 34681158 10.3390/biology10101061 PMC8533319

[13] Whitton BA, Potts M, editors. The ecology of cyanobacteria: their diversity in time and space. Dordrecht: Kluwer Academic; 2002. 10.1046/j.1366-9516.2001.00118.x.

[14] Chittora D, Meena M, Barupal T, Swapnil P, Sharma K. Cyanobacteria as a source of biofertilizers for sustainable agriculture. Biochem Biophys Rep. 2020;22:100737. 10.1016/j.bbrep.2020.100737. 32083191 10.1016/j.bbrep.2020.100737 PMC7021550

[15] Singh JS, Kumar A, Rai AN, Singh DP. Cyanobacteria. A precious bio-resource in agriculture, ecosystem, and environmental sustainability. Front Microbiol. 2016;7:529. 10.3389/fmicb.2016.00529. 27148218 10.3389/fmicb.2016.00529 PMC4838734

[16] Bello AS, Ben-Hamadou R, Hamdi H, Saadaoui I, Ahmed T. Application of cyanobacteria (*Roholtiella* sp.) liquid extract for the alleviation of salt stress in bell pepper (*Capsicum annuum* L.) plants grown in a soilless system. Plants. 2022;11:104. 10.3390/plants11010104. 10.3390/plants11010104 PMC8747557 35009109

[17] Mutale-joan C, Redouane B, Najib E, et al. Screening of microalgae liquid extracts for their bio stimulant properties on plant growth, nutrient uptake and metabolite profile of *Solanum lycopersicum* L. Sci Rep. 2020;10:2820. 10.1038/s41598-020-59840-4. 32071360 10.1038/s41598-020-59840-4 PMC7028939

[18] Buss W, Belt K, Hein Z, Putri A, Zhu S, Suwandari H, Bentle AR. Hurdles to overcome to achieve biostimulant-driven, low chemical input crop production. Plants People Planet. 2025. 10.1002/ppp3.70030.

[19] Yakhin OI, Lubyanov AA, Yakhin IA, Brown PH. Biostimulants in plant science: a global perspective. Front Plant Sci. 2017;7:2049. 10.3389/fpls.2016.02049. 28184225 10.3389/fpls.2016.02049 PMC5266735

[20] Mughunth RJ, Velmurugan S, Mohanalakshmi M, Vanitha K. A review of seaweed extract’s potential as a biostimulant to enhance growth and mitigate stress in horticulture crops. Sci Hortic. 2024;334:113312. 10.1016/j.scienta.2024.113312.

[21] Nawaz T, Saud S, Gu L, Khan I, Fahad S, Zhou R, Cyanobacteria. Harnessing the power of microorganisms for plant growth promotion, stress alleviation, and phytoremediation in the era of sustainable agriculture. Plant Stress. 2024;11:100399. 10.1016/j.stress.2024.100399.

[22] Taiz L, Zeiger E. (2010) Plant Physiology. 5th Edition, Sinauer Associates Inc., Sunderland, 782 p.

[23] Santini G, Biondi N, Rodolfi L, Tredici MR. Plant biostimulants from cyanobacteria: an emerging strategy to improve yields and sustainability in agriculture. Plants. 2021;10:643DOI. 10.3390/plants10040643. 33805266 10.3390/plants10040643 PMC8065465

[24] Rossi F, De Philippis R. Role of cyanobacterial exopolysaccharides in phototrophic biofilms and in complex microbial mats. Life. 2015;5:1218–38. 10.3390/life5021218. 25837843 10.3390/life5021218 PMC4500136

[25] Bhagat N, Raghav M, Dubey S, Bedi N. Bacterial exopolysaccharides: insight into their role in plant abiotic stress tolerance. 2021;31:1045–59. 10.4014/jmb.2105.05009 10.4014/jmb.2105.05009 PMC9706007 34226402

[26] Parwani L, Bhatt M, Singh J. Potential biotechnological applications of cyanobacterial exopolysaccharides. Braz Arch Biol Technol. 2021;64:e21200401. 10.1590/1678-4324-2021200401.

[27] Robati SMS. Cyanobacteria: A Promising Future for Sustainable Agriculture. In: Severo IA, Martínez-Burgos WJ, Ordonez J, editors. Insights Into Algae - Fundamentals, Culture Techniques and Biotechnological Uses of Microalgae and Cyanobacteria. IntechOpen; 2024.10.5772/intechopen.1005021

[28] Santini G, Biondi N, Rodolfi L, Tredici MR. Plant biostimulants from cyanobacteria: an emerging strategy to improve yields and sustainability in agriculture. Plants. 2021;10:643. 10.3390/plants10040643. 33805266 10.3390/plants10040643 PMC8065465

[29] Poveda J. Cyanobacteria in plant health: biological strategy against abiotic and biotic stresses. Crop Prot. 2021;141:105450. 10.1016/j.cropro.2020.105450.

[30] Singh S. A review on possible elicitor molecules of cyanobacteria: their role in improving plant growth and providing tolerance against biotic or abiotic stress. J Appl Microbiol. 2014;117:1221–44. 10.1111/jam.12612. 25069397 10.1111/jam.12612

[31] Goiris K, Muylaert K, Voorspoels S, Noten B, De Paepe D, Baart E. Detection of flavonoids in microalgae from different evolutionary lineages. J Phycol. 2014;50:483–92. 10.1111/jpy.12180. 26988321 10.1111/jpy.12180

[32] Gill SS, Tuteja N. Reactive oxygen species and antioxidant machinery in abiotic stress tolerance in crop plants. Plant Physiol Biochem. 2010;48:909–30. 10.1016/j.plaphy.2010.08.016. 20870416 10.1016/j.plaphy.2010.08.016

[33] Guerreiro A, Andrade MA, Menezes C, Vilarinho F, Dias E. Antioxidant and cytoprotective properties of cyanobacteria: potential for biotechnological applications. Toxins. 2020;12:548. 10.3390/toxins12090548. 32859010 10.3390/toxins12090548 PMC7551995

[34] Shawer E, Elsaied H, El-Gamal A, Sabae S. Characterization of cyanobacterial isolates from freshwater and saline subtropical desert lakes. Folia Microbiol (Praha). 2023;68:403–14. 10.1007/s12223-022-01016-w. 36504332 10.1007/s12223-022-01016-w PMC10205856

[35] Vaz MGMV, Genuário DB, Andreote APD, Malone CFS, Sant’Anna CL, Barbiero L, et al. *Pantanalinema* gen. nov. and *Alkalinema* gen. nov.: novel pseudanabaenacean genera (Cyanobacteria) isolated from saline–alkaline lakes. Int J Syst Evol Microbiol. 2015;65(Pt_1):298–308. 10.1099/ijs.0.070110-0. 25351877 10.1099/ijs.0.070110-0

[36] Pade N, Hagemann M. Salt acclimation of cyanobacteria and their application in biotechnology. Life. 2015;5:25–49. 10.3390/life5010025. 10.3390/life5010025 PMC4390839 25551682

[37] Yadav P, Singh RP, Rana S, Joshi D, Kumar D, Bhardwaj N, et al. Mechanisms of stress tolerance in cyanobacteria under extreme conditions. Stresses. 2022;2:531–49. 10.3390/stresses2040036.

[38] Shewry PR, Hey SJ. The contribution of wheat to human diet and health. Food Energy Secur. 2015;4:178–202. 10.1002/fes3.64. 27610232 10.1002/fes3.64 PMC4998136

[39] Negacz K, Malek Ž, de Vos A, Vellinga P. Saline soils worldwide: identifying the most promising areas for saline agriculture. J Arid Environ. 2022;203:104775. 10.1016/j.jaridenv.2022.104775.

[40] Nicolas F, Kamai T, Ben-Gal A, Ochoa-Brito J, Daccache A, Ogunmokun F, et al. Assessing salinity impacts on crop yield and economic returns in the central Valley. Agric Water Manage. 2023;287:108463. 10.1016/j.agwat.2023.108463.

[41] Al-Ashkar I, Alderfasi A, Ben Romdhane W, Seleiman MF, El-Said RA, Al-Doss A. Morphological and genetic diversity within salt tolerance detection in eighteen wheat genotypes. Plants. 2020;9:287. 10.3390/plants9030287. 32106488 10.3390/plants9030287 PMC7154827

[42] Sellami MH, Di Mola I, Mori M. Evaluating wheat response to biostimulants: a 25-year review of field-based research (2000–2024). Front Sustain Food Syst. 2025;9:1543981. 10.3389/fsufs.2025.1543981.

[43] Casamatta DA, Johansen JR, Vis ML, Broadwater ST. Molecular and morphological characterization of ten polar and near-polar strains within the Oscillatoriales (cyanobacteria). J Phycol. 2005;41:421–38. 10.1111/j.1529-8817.2005.04062.x.

[44] Kimura M. A simple method for estimating evolutionary rates of base substitutions through comparative studies of nucleotide sequences. J Mol Evol. 1980;16:111–20. 10.1007/BF01731581. 7463489 10.1007/BF01731581

[45] Kumar K, Stecher G, Suleski M, Sanderford M, Sharma S, Tamura K. MEGA12: Molecular Evolutionary Genetic Analysis Version 12 for Adaptive and Green Computing, *Molecular Biology and Evolution*, Volume 41, Issue 12, December 2024, msae263. 10.1093/molbev/msae263 10.1093/molbev/msae263 PMC11683415 39708372

[46] Rady MM, Boriek SHK, Abd El-Mageed TA, Seif El-Yazal MA, Ali EF, Hassan FAS, et al. Exogenous gibberellic acid or dilute bee honey boosts drought stress tolerance in *Vicia faba* by rebalancing osmoprotectants, antioxidants, nutrients, and phytohormones. Plants. 2021;10:748. 10.3390/plants10040748. 10.3390/plants10040748 PMC8068922 33920494

[47] Semida WM, Abdelkhalik A, Mohamed GF, Abd El-Mageed TA, Abd El-Mageed SA, Rady MM, et al. Foliar application of zinc oxide nanoparticles promotes drought stress tolerance in eggplant (*Solanum melongena* L). Plants. 2021;10:421. 10.3390/plants10020421. 33672429 10.3390/plants10020421 PMC7926631

[48] Agami RA. Pre-soaking in indole-3-acetic acid or spermidine enhances copper tolerance in wheat seedlings. South Afr J Bot. 2016;104:167–74. 10.1016/j.sajb.2015.10.003.

[49] Mohamed IAA, Shalby N, El-Badri MA, Saleem A, Khan MH, Nawaz MNA. Stomata and xylem vessels traits improved by melatonin application contribute to enhancing salt tolerance and fatty acid composition of *Brassica napus* L. plants. Agronomy. 2020;10:1186. 10.3390/agronomy10081186.

[50] Sass JE, UK. Botanical microtechnique. 3rd edition. USA; : The Iowa State College Press; Constable & Co; 1958. 10.31274/isudp.25

[51] Mohamed IAA, Shalby N, Bai C, Qin M, Agami RA, Jie K, et al. Stomatal and photosynthetic traits are associated with investigating sodium chloride tolerance of *Brassica napus* L. cultivars. Plants. 2020;9:62. 10.3390/plants9010062. 10.3390/plants9010062 PMC7020420 31906529

[52] Ubeda-Tomás S, Federici F, Casimiro I, Beemster GTS, Bhalerao R, Swarup R, et al. Gibberellin signaling in the endodermis controls Arabidopsis root meristem size. Curr Biol. 2009;19:1194–9. 10.1016/j.cub.2009.06.023. 19576770 10.1016/j.cub.2009.06.023

[53] Cheng P, Liu P, Cao L, Wang S, Li P, Zhang P, et al. Down-regulation of multiple CDK inhibitor ICK/KRP genes promotes cell proliferation, callus induction and plant regeneration in Arabidopsis. Front Plant Sci. 2015;6:825. 10.3389/fpls.2015.00825. 26528298 10.3389/fpls.2015.00825 PMC4602110

[54] Xu P, Wang S, Tang L, Wang P, Li Z, Wang P. Differential influence of cortex and stele components on root tip diameter in different types of tropical climbing plants. Front Plant Sci. 2022;13:961214. 10.3389/fpls.2022.961214. 36119575 10.3389/fpls.2022.961214 PMC9470880

[55] Lüdecke D, Ben-Shachar M, Patil I, Waggoner P, Makowski D. Performance: an R package for assessment, comparison and testing of statistical models. JOSS. 2021;6:3139. 10.21105/joss.03139.

[56] Majeed A, Muhammad Z, Salinity. A major agricultural Problem-Causes, impacts on crop productivity and management strategies. In: Hasanuzzaman M, Hakeem KR, Nahar K, Alharby HF, editors. Plant abiotic stress tolerance: agronomic, molecular and biotechnological approaches. Cham: Springer International Publishing; 2019. pp. 83–99. 10.1007/978-3-030-06118-0_3.

[57] Abdellaoui R, Elkelish A, El-Keblawy A, Mighri P, Boughalleb F, Bakhshandeh E, Editorial. Halophytes: salt stress tolerance mechanisms and potential use. Front Plant Sci. 2023;14:1218184. 10.3389/fpls.2023.1218184. 37426981 10.3389/fpls.2023.1218184 PMC10325650

[58] Muhammad M, Waheed A, Wahab A, Majeed M, Nazim M, Liu P, et al. Soil salinity and drought tolerance: an evaluation of plant growth, productivity, microbial diversity, and amelioration strategies. Plant Stress. 2024;11:100319. 10.1016/j.stress.2023.100319.

[59] Calvo P, Nelson L, Kloepper JW. Agricultural uses of plant biostimulants. Plant Soil. 2014;383:3–41. 10.1007/s11104-014-2131-8.

[60] Hamouda RA, Shehawy MA, Din SMME, Albalwe FM, Albalawi HMR, Hussein MH. Protective role of *Spirulina platensis* liquid extract against salinity stress effects on *Triticum aestivum* L. Green Process Synth. 2022;11:648–58. 10.1515/gps-2022-0065.

[61] Fiorentino S, Bellani L, Santin M, Castagna A, Echeverria MC, Giorgetti L. Effects of microalgae as biostimulants on plant growth, content of antioxidant molecules and total antioxidant capacity in *Chenopodium quinoa* exposed to salt stress. Plants. 2025;14:781. 10.3390/plants14050781. 40094757 10.3390/plants14050781 PMC11902087

[62] Arif P, Singh P, Siddiqui P, Bajguz A, Hayat S. Salinity induced physiological and biochemical changes in plants: an omic approach towards salt stress tolerance. Plant Physiol Biochem. 2020;156:64–77. 10.1016/j.plaphy.2020.08.042. 32906023 10.1016/j.plaphy.2020.08.042

[63] Johnson R, Puthur JT. Seed priming as a cost effective technique for developing plants with cross tolerance to salinity stress. Plant Physiol Biochem. 2021;162:247–57. 10.1016/j.plaphy.2021.02.034. 33711718 10.1016/j.plaphy.2021.02.034

[64] Crouch IJ, Van Staden J. Evidence for the presence of plant growth regulators in commercial seaweed products. Plant Growth Regul. 1993;13:21–9. 10.1007/BF00207588.

[65] El-Dahma AM, Ahmed EA, Abd El-Sadek ME. An innovative use of microalgal extracts as an alternative to expensive growth regulators via biotechnological techniques. Egypt J Bot. 2024;64:219–33. 10.21608/ejbo.2024.278921.2773.

[66] Supraja KV, Behera B, Balasubramanian P. Efficacy of microalgal extracts as biostimulants through seed treatment and foliar spray for tomato cultivation. Ind Crops Prod. 2020;151:112453. 10.1016/j.indcrop.2020.112453.

[67] Inamuddin, Ahamed MI, Boddula R, Rezakazemi M, editors. Biofertilizers: study and impact. Beverley, MA: Scrivener Publishing; 2021.

[68] Fathy WA, AbdElgawad P, Hashem AH, Essawy E, Tawfik E, Al-Askar AA, et al. Exploring exogenous Indole-3-acetic acid’s effect on the growth and biochemical profiles of *Synechocystis* sp. PAK13 and *Chlorella**variabilis*. Molecules. 2023;28:5501. 10.3390/molecules28145501. 10.3390/molecules28145501 PMC10385099 37513371

[69] Sergeeva E, Liaimer A, Bergman B. Evidence for production of the phytohormone indole-3-acetic acid by cyanobacteria. Planta. 2002;215:229–38. 10.1007/s00425-002-0749-x. 12029472 10.1007/s00425-002-0749-x

[70] Wang C, Qi M, Guo J, Zhou C, Yan X, Ruan R, et al. The active phytohormone in microalgae: the characteristics, efficient detection, and their adversity resistance applications. Molecules. 2022;27:46. 10.3390/molecules27010046. 10.3390/molecules27010046 PMC8746318 35011277

[71] Afzal Z, Howton TC, Sun P, Mukhtar MS. The roles of Aquaporins in plant stress responses. J Dev Biol. 2016;4:9. 10.3390/jdb4010009. 29615577 10.3390/jdb4010009 PMC5831814

[72] Nie P, Gong B, Wen D, Qiao P, Guo P, Shi Q. Brassinosteroid enhances cucumber stress tolerance to NaHCO3 by modulating nitrogen metabolism, ionic balance and phytohormonal response. Plants. 2025;14:80. 10.3390/plants14010080. 10.3390/plants14010080 PMC11723003 39795340

[73] Zhou P, Shi P, Yang P, Feng X, Chen X, Xiao F, et al. Insights into plant salt stress signaling and tolerance. J Genet Genomics. 2024;51:16–34. 10.1016/j.jgg.2023.08.007. 37647984 10.1016/j.jgg.2023.08.007

[74] Ashraf M, Harris PJC. Photosynthesis under stressful environments: an overview. Photosynthetica. 2013;51:163–90. 10.1007/s11099-013-0021-6.

[75] Godlewska K, Michalak I, Tuhy Ł, Chojnacka K. Plant growth biostimulants based on different methods of seaweed extraction with water. BioMed Res Int. 2016;5973760. 10.1155/2016/5973760. 27366749 10.1155/2016/5973760 PMC4913069

[76] Bhupenchandra I, Chongtham SK, Devi EL, Choudhary RR, Salam AK. Role of biostimulants in mitigating the effects of climate change on crop performance. Front Plant Sci. 2022;13. 10.3389/fpls.2022.967665. 10.3389/fpls.2022.967665 PMC9634556 36340395

[77] Di Sario L, Boeri P, Matus JT, Pizzio GA. Plant biostimulants to enhance abiotic stress resilience in crops. Int J Mol Sci. 2025;26(3):1129. 10.3390/ijms26031129. 39940896 10.3390/ijms26031129 PMC11817731

[78] Shabala S, Munns R. Salinity stress: physiological constraints and adaptive mechanisms. In: Plant stress physiology. 2017. pp. 24–63. 10.1079/9781780647296.0024

[79] Acosta-Motos JR, Ortuño MF, Bernal-Vicente A, Diaz-Vivancos P, Sanchez-Blanco MJ, Hernandez JA. Plant responses to salt stress: adaptive mechanisms. Agronomy. 2017;7:18. 10.20944/preprints201702.0083.v2.

[80] Farooq M, Rafique S, Zahra N, Rehman A, Siddique KHM. Root system architecture and salt stress responses in cereal crops. J Agron Crop Sci. 2024;210:e12776. 10.1111/jac.12776.

[81] Ivanchenko MG, Napsucialy-Mendivil S, Dubrovsky JG. Auxin-induced Inhibition of lateral root initiation contributes to root system shaping in *Arabidopsis**thaliana*. Plant J. 2010;64:740–52. 10.1111/j.1365-313X.2010.04365.x. 10.1111/j.1365-313X.2010.04365.x 21105922

[82] Overvoorde P, Fukaki P, Beeckman T. Auxin control of root development. Cold Spring Harb Perspect Biol. 2010;2:a001537. 10.1101/cshperspect.a001537. 20516130 10.1101/cshperspect.a001537 PMC2869515

[83] Abd El Baky HH, Hussein MM, El-Baroty GS. Induces of antioxidant compounds and salttolerance in wheat plant, irrigated with seawater as response to application of micro algae spry. AJABS. 2014;9:127–37. 10.3844/ajabssp.

[84] Gupta RC, editor. Handbook of toxicology of chemical warfare agents. Third edition. London, United Kingdom; San Diego, CA: Academic Press, an imprint of Elsevier; 2020. 10.1016/B978-0-12-374484-5.X0001-6

[85] Kieber JJ, Schaller GE, Cytokinins. Arabidopsis Book. 2014;12:e0168. 10.1199/tab.0168. 24465173 10.1199/tab.0168 PMC3894907

[86] Cheng S, Wang Q, Manghwar P, Liu F. Autophagy-Mediated regulation of different meristems in plants. Int J Mol Sci. 2022;23:6236. 10.3390/ijms23116236. 35682913 10.3390/ijms23116236 PMC9180974

[87] Hayashi K, Hasegawa J, Matsunaga S. The boundary of the meristematic and elongation zones in roots: endoreduplication precedes rapid cell expansion. Sci Rep. 2013;3:2723. 10.1038/srep02723. 24121463 10.1038/srep02723 PMC3796303

[88] Shukla PS, Shotton K, Norman E, Neily P, Critchley AT, Prithiviraj B. Seaweed extract improve drought tolerance of soybean by regulating stress-response genes. AoB Plants. 2017;10. 10.1093/aobpla/plx051. 10.1093/aobpla/plx051 PMC5751077 29308122

[89] Fal S, Aasfar A, Rabie R, Smouni A, Arroussi HEL. Salt induced oxidative stress alters physiological, biochemical and metabolomic responses of green microalga *Chlamydomonas reinhardtii*. Heliyon. 2022;8:e08811. 10.1016/j.heliyon. 35118209 10.1016/j.heliyon.2022.e08811 PMC8792077

[90] Tavu LEJ, Redillas MCFR. Oxidative stress in rice (*Oryza sativa*): mechanisms, impact, and adaptive strategies. Plants. 2025;14:1463. 10.3390/plants14101463. 40431027 10.3390/plants14101463 PMC12114693

[91] Oluwole O, Fernando WB, Lumanlan J, Ademuyiwa O, Jayasena V. Role of phenolic acid, tannins, stilbenes, lignans and flavonoids in human health-a review. Int J Food Sci Tech. 2022;57:6326–35. 10.1111/ijfs.15936.

[92] Volpi e Silva N, Mazzafera P, Cesarino I. Should I stay or should I go: are chlorogenic acids mobilized towards lignin biosynthesis? Phytochemistry. 2019;166:112063. 10.1016/j.phytochem. 31280091 10.1016/j.phytochem.2019.112063

[93] Pasternak TP, Steinmacher D. Plant growth regulation in cell and tissue culture. Vitro Plants. 2024;13:327. 10.3390/plants13020327. 38276784 10.3390/plants13020327 PMC10818547

[94] Sosnowski J, Truba M, Vasileva V. The impact of auxin and cytokinin on the growth and development of selected crops. Agriculture. 2023;13:724. 10.3390/agriculture13030724.

[95] Gao J, Zhuang S, Zhang P. Advances in plant auxin biology: synthesis, metabolism, signaling, interaction with other hormones, and roles under abiotic stress. Plants. 2024;13:2523. 10.3390/plants13172523. 39274009 10.3390/plants13172523 PMC11397301

[96] Karahara I, Horie T. Functions and structure of roots and their contributions to salinity tolerance in plants. Breed Sci. 2021;71:89–108. 10.1270/jsbbs.20123. 33762879 10.1270/jsbbs.20123 PMC7973495

[97] Delgado C, Mora-Poblete F, Ahmar S, Chen J-T, Figueroa CR. Jasmonates and plant salt stress: molecular players, physiological effects, and improving tolerance by using genome-associated tools. Int J Mol Sci. 2021;22:3082. 10.3390/ijms22063082. 33802953 10.3390/ijms22063082 PMC8002660

[98] Natarajan PN, Singh S, Balamurugan K. Gas chromatography-mass spectrometry (GC-MS) analysis of bio active compounds presents in *Oeophylla smaragdina*. Res J Pharm Technol. 2019;12(6):2736–41. 10.5958/0974-360X.2019.00458.X.

[99] Santhiya N, Ramasamy M. GC-MS analysis of bioactive compounds from freshwater mussels of *parreysia corrugata* (Muller 1774) and their pharmacological activities. J Drug Deliv Ther. 2019;9:155–8. 10.22270/jddt.v9i4-A.3447.

[100] Thamer FH, Thamer N. Gas chromatography – mass spectrometry (GC-MS) profiling reveals newly described bioactive compounds in *Citrullus colocynthis* (L.) seeds oil extracts. Heliyon. 2023. 10.1016/j.heliyon.2023.e16861. 10.1016/j.heliyon.2023.e16861 PMC10360964 37484228

[101] Ali O, Szabó A. Review of eukaryote cellular membrane lipid composition, with special attention to the fatty acids. Int J Mol Sci. 2023;24:15693. 10.3390/ijms242115693. 37958678 10.3390/ijms242115693 PMC10649022

[102] Krajciova D, Holic R. The plasma membrane H+-ATPase promoter driving the expression of FADX enables highly efficient production of punicic acid in *Rhodotorula toruloides* cultivated on glucose and crude glycerol. J Fungi. 2024;10:649. 10.3390/jof10090649. 10.3390/jof10090649 PMC11433134 39330409

[103] Taybi T, Alyahya N. Comparative analysis of physiological and biochemical responses to salt stress reveals important mechanisms of salt tolerance in wheat. Int J Mol Sci. 2025;26:3742. 10.3390/ijms26083742. 40332384 10.3390/ijms26083742 PMC12028197

[104] Lindberg S, Premkumar A. Ion changes and signaling under salt stress in wheat and other important crops. Plants. 2024;13:46. 10.3390/plants13010046. 10.3390/plants13010046 PMC10780558 38202354

[105] Shaari NEM, Khandaker MM, Tajudin MTFM, Majrashi A, Alenazi MM, Bakar MFA, et al. Effects of *Spirulina platensis* and *Sargassum polycystum* extracts on morpho-physiological and metabolic responses of Pak Choi under cadmium stress. J Appl Phycol. 2025. 10.1007/s10811-025-03570-5.

[106] Solano C, Artola A, Barrena R, Ballardo C, Sánchez A. Effect of the exogenous application of different concentrations of Indole-3-acetic acid as a growth regulator on onion (*Allium cepa* L.) cultivation. Agronomy. 2023;13:2204. 10.3390/agronomy13092204.

